# Differential gene expression analysis identified determinants of cell fate plasticity during radiation-induced regeneration in *Drosophila*

**DOI:** 10.1371/journal.pgen.1009989

**Published:** 2022-01-06

**Authors:** Michelle Ledru, Caitlin A. Clark, Jeremy Brown, Shilpi Verghese, Sarah Ferrara, Andrew Goodspeed, Tin Tin Su

**Affiliations:** 1 Department of Molecular, Cellular and Developmental Biology, University of Colorado, Boulder, Colorado, United States of America; 2 University of Colorado Cancer Center, Anschutz Medical Campus, Aurora, Colorado, United States of America; 3 Department of Pharmacology, University of Colorado Anschutz Medical Campus, Aurora, Colorado, United States of America; Geisel School of Medicine at Dartmouth, UNITED STATES

## Abstract

Ionizing radiation (IR) is used to treat half of all cancer patients because of its ability to kill cells. IR, however, can induce stem cell-like properties in non-stem cancer cells, potentiating tumor regrowth and reduced therapeutic success. We identified previously a subpopulation of cells in *Drosophila* larval wing discs that exhibit IR-induced stem cell-like properties. These cells reside in the future wing hinge, are resistant to IR-induced apoptosis, and are capable of translocating, changing fate, and participating in regenerating the pouch that suffers more IR-induced apoptosis. We used here a combination of lineage tracing, FACS-sorting of cells that change fate, genome-wide RNAseq, and functional testing of 42 genes, to identify two key changes that are required cell-autonomously for IR-induced hinge-to-pouch fate change: (1) repression of hinge determinants Wg (*Drosophila* Wnt1) and conserved zinc-finger transcription factor Zfh2 and (2) upregulation of three ribosome biogenesis factors. Additional data indicate a role for Myc, a transcriptional activator of ribosome biogenesis genes, in the process. These results provide a molecular understanding of IR-induced cell fate plasticity that may be leveraged to improve radiation therapy.

## Author summary

Ionizing radiation (IR) is used to treat half of all cancer patients because of its ability to kill cells but treatment failures are common because tumors grow back (regenerate). Here, we asked which changes in the properties of cells facilitate regeneration in *Drosophila* (fruit flies) after exposure to radiation. We identified six genes whose products increase or decrease the regenerative potential of cells. These results help us understand how tissues regenerate after IR damage and will aid in designing better therapies that involve radiation.

## Introduction

More than half of cancer patients receive ionizing radiation (IR), alone or as a component of their treatment (www.cancer.org). IR induces DNA damage to kill cells. Surviving cancer cells could, however, regenerate the tumor, leading to treatment failure. Regeneration of tumors is attributed to cancer cells with stem cell-like properties [1,2]. These cells, distinguished by cancer type-specific markers such as CD44 and ALDH for Head and Neck Squamous Cell Carcinoma, survive treatment and show elevated capacity to initiate tumors compared to their counterparts that lack the markers [3,4]. Identifying and eliminating cancer stem-like cells is a goal for improved therapy. A body of literature shows that the proportion of cancer cells with stem cell markers increases after treatment [5], and that in some cases, the treatment itself such as IR is found to induce stemness [6–9]. There is also evidence for non-stem cells acquiring stem cell-like properties in the context of regeneration of normal tissues after radiation damage. In irradiated mouse intestine, Paneth cells de-differentiate to populate the stem cell compartment [10]; forced activation of Notch signaling recapitulates this process in the absence of irradiation. In irradiated mouse salivary glands, acinar and duct cells adopt plasticity to regenerate acinar cells [11]. Thus, there is mounting evidence that IR can induce cell fate plasticity, but the mechanisms remain to be fully understood.

Regeneration of *Drosophila* larval organs called imaginal discs occurs without a dedicated stem cell pool. We identified a previously unknown mode of regeneration in *Drosophila* larval wing discs whereby epithelial cells acquire stem cell-like properties after irradiation [12–14]. These properties include the ability to change cell fate and translocate to areas of the disc with greater need for cell replenishment. The ability to behave like stem cells, we found, is induced by IR and is limited to a specific subset of cells within the wing disc, those of the future hinge that connects the wing blade to the body.

The hinge region of the wing disc displays unique features. It experiences a combination of high Wingless (Wg, *Drosophila* Wnt1) and Stat92E (*Drosophila* STAT3/5) signals, which act together to promote the growth and differentiation of the region during normal development into a structure that connects the wing blade to the body wall in the adult [14]. In the larval wing disc, hinge cells show different cytoskeletal and extracellular matrix organization than the rest of the disc and are highly tumorigenic; they undergo neoplastic transformation under conditions that have little effect on the rest of the wing disc such as mutations in tumor suppressors *lgl* and *scrib*, leading to the hinge being called a ‘tumor hotspot’ [15]. When the adjacent pouch region is genetically ablated by localized ectopic expression of pro-apoptotic genes, the cells of the hinge change fate to regenerate the pouch [16]. We have shown that hinge cells are resistant to IR-induced apoptosis and that this protection depends on Wg and Stat92E [14]. Importantly, irradiated hinge cells lose the hinge fate as seen by the loss of expression of hinge markers, gain pouch fate as seen by the gain of expression of a pouch marker, translocate to the pouch and help regenerate the latter. Candidate testing identified apoptotic caspases and cytoskeletal proteins as important for hinge-to-pouch conversion [12,17], but we lack a comprehensive understanding of this fate change process.

Here, we performed a genome-wide analysis of gene expression changes during regeneration of the *Drosophila* larval wing disc. By dissociating the discs into single cells and using Fluorescence-Activated Cell Sorting (FACS) we monitored the hinge versus the rest of the disc, to identify genes that are differentially expressed in the two cell populations during regeneration and fate change. Functional testing of 42 candidate regulators identified six genes whose experimental manipulation compromised hinge-to-pouch conversion. These six genes encode hinge determinants (Zfh2 and Wg) or ribosome biogenesis factors (RpI135, Rs1, Tsr1 and Myc), suggesting that cell fate plasticity during regeneration in irradiated wing discs requires downregulation of transcripts for hinge determinants and concomitant upregulation of translation capacity. These results illustrate that cell fate change requires much more than simply transcribing genes for the new fate; suppression of old fate determinants and post-transcriptional mechanisms that generate new fate determinants appear equally important.

## Results

### Characterization of hinge-specific 30A-GAL4 driver

**Fig 1:**
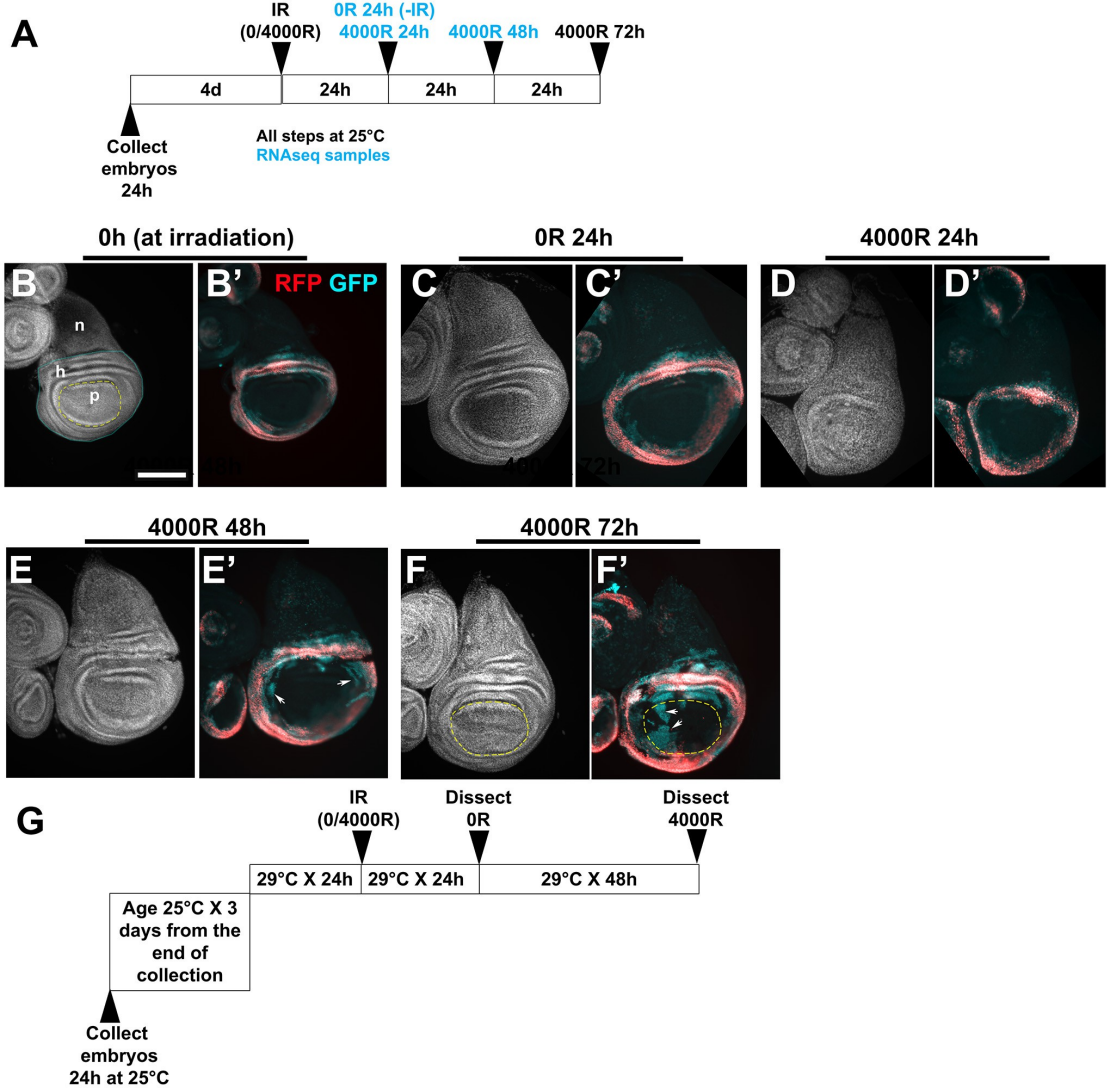
Lineage tracing to monitor cell fate changes after irradiation. Larvae expressing G-trace under the control of 30A-GAL4 were irradiated with 0 or 4000R of X-rays. The discs were dissected at various times shown after irradiation, fixed, stained for DNA and imaged for DNA, RFP and GFP. The larvae were of the genotype w^1118^/+ or Y; 30A-GAL4>UAS-G-trace/+ produced by a cross between w^1118^ and 30A-GAL4>UAS-G-trace/SM5 and sorted for RFP/GFP. Scale bar = 100 microns. (A) The protocol used to generate the samples for RNAseq. (B-F) Representative discs from various time points. (B-B’) show discs at the time of irradiation. Note the overlap of RFP and GFP, indicating little fate plasticity, and the absence of GFP^+^ cells in the pouch within the yellow line in B. DNA images are used to discern the different regions of the disc; n = notum, h = hinge, p = pouch. (C-C’) show an unirradiated disc at 24h after mock irradiation, the time point for -IR RNAseq samples. (D-D’) show an irradiated disc at 24h after IR, showing little fate change. The IR+24h RNAseq samples were from such discs. (E-E’) show an irradiated disc at 48h after IR, showing the first indications of fate change as seen by RFP^-^GFP^+^ cells (arrows). Similar results are seen also in larvae that were irradiated 3 days from the end of egg collection. The IR+48h RNAseq samples included larvae irradiated 3 or 4 days from the end of egg collection. (F-F’) show an example of a disc with fate change as indicated by RFP^-^GFP^+^ cells (arrows) in the pouch (yellow circle). Such fate change was seen at the IR+72h time point when larvae were irradiated 3d (shown here) or 4d from the end of egg collection [14]. (G) The temperature shift and irradiation protocol used in the functional tests. The temperature shift inactivates GAL80^ts^ that was present in the crosses for the functional tests.

To lineage-trace hinge cells, we used a hinge-specific 30A-GAL4 driver, whose expression is entirely within the hinge region as defined by antibody staining for the hinge determinant Zfh2 [12]. We mapped 30A-GAL4 by plasmid rescue to 21E2, at genomic location 2L:685363. 685363[+] (Release 5.28 of *D*. *melanogaster* genome), within an intron of the *dachsous* gene that encodes a member of the cadherin family ([18] gene nomenclature follows FlyBase). *ds* is expressed more broadly in the wing disc than 30A-GAL4 [18], suggesting that 30A recapitulates only a subset of ds expression. More important, expression of the dual-color G-trace reporter in which RFP is the real-time maker and GFP is the lineage tracer [19] showed that cell fates are stable in the 30A-GAL4 domain as indicated by RFP/GFP overlap ([12–14]; reproduced in Fig 1B and 1B’). After irradiation, however, some hinge cells marked with GFP lose hinge-specific 30A-GAL4 activity (become RFP^-^) and translocate to the pouch region ([12–14]; Fig 1F and 1F’ arrows). We interpret RFP^+^GFP^+^ cells as those in the hinge and RFP^-^GFP^+^ cells as those that originated in the hinge but lost the hinge identity.

We have shown previously that fate change and translocation commence at about 48h after exposure to 4000R of X-rays [14]. We reproduce this result here. RFP^-^GFP^+^ cells are scarce at 24h after irradiation (Fig 1D’) but appear by 48h after irradiation (Fig 1E’ arrows). Fate change is maximal at 72h after irradiation such that RFP^-^GFP^+^ cells are found in the pouch (Fig 1F’ arrows; yellow line indicates the pouch). We lose the larvae to pupariation at later times after IR. This schedule of hinge-to-pouch conversion was observed whether we irradiated larvae 3 days from the end of egg collection [13] or 4 days from the end of egg collection [14]. We showed previously that RFP^-^GFP^+^ cells in the pouch have not only lost the hinge fate (became RFP^-^) but also have acquired the pouch fate as detected by VgE-lacZ expression [12]. To understand the molecular changes that regulate fate change and translocation, we analyzed gene expression at 24 and 48h after irradiation and at 24h without irradiation. These earlier time points, we reasoned, are more likely to capture changes that cause fate change/translocation by 72h after IR. The 72h time point was used in functional testing of candidate regulators of fate change described later, with a temperature shift in the protocol to activate GAL4 conditionally (Fig 1G).

### IR-induced gene expression changes in the hinge versus the rest of the disc

To capture IR-induced gene expression changes specifically in the hinge, the discs were dissociated into single cells and sorted into RFP^+^GFP^+^ (hinge) and RFP^-^GFP^-^ pools (FACS profile in S1 Fig). The latter group would include cells of the pouch and the notum. We detected RFP^-^GFP^+^ cells as well, but these were not included in the analysis for two reasons. First, we reasoned that they have already changed fate and therefore were beyond the state of interest. Second, there were too few RFP^-^GFP^+^ cells at the time points of interest, 24 and 48h after IR, for meaningful analysis. Thus, we compared 6 samples: RFP^+^GFP^+^ (POS or pos) and RFP^-^GFP^-^ (NEG or neg) for each of the three time points, -IR, IR+24h and IR+48h. Each sample was acquired in duplicate to achieve two biological replicates of the whole experiment.

**Fig 2:**
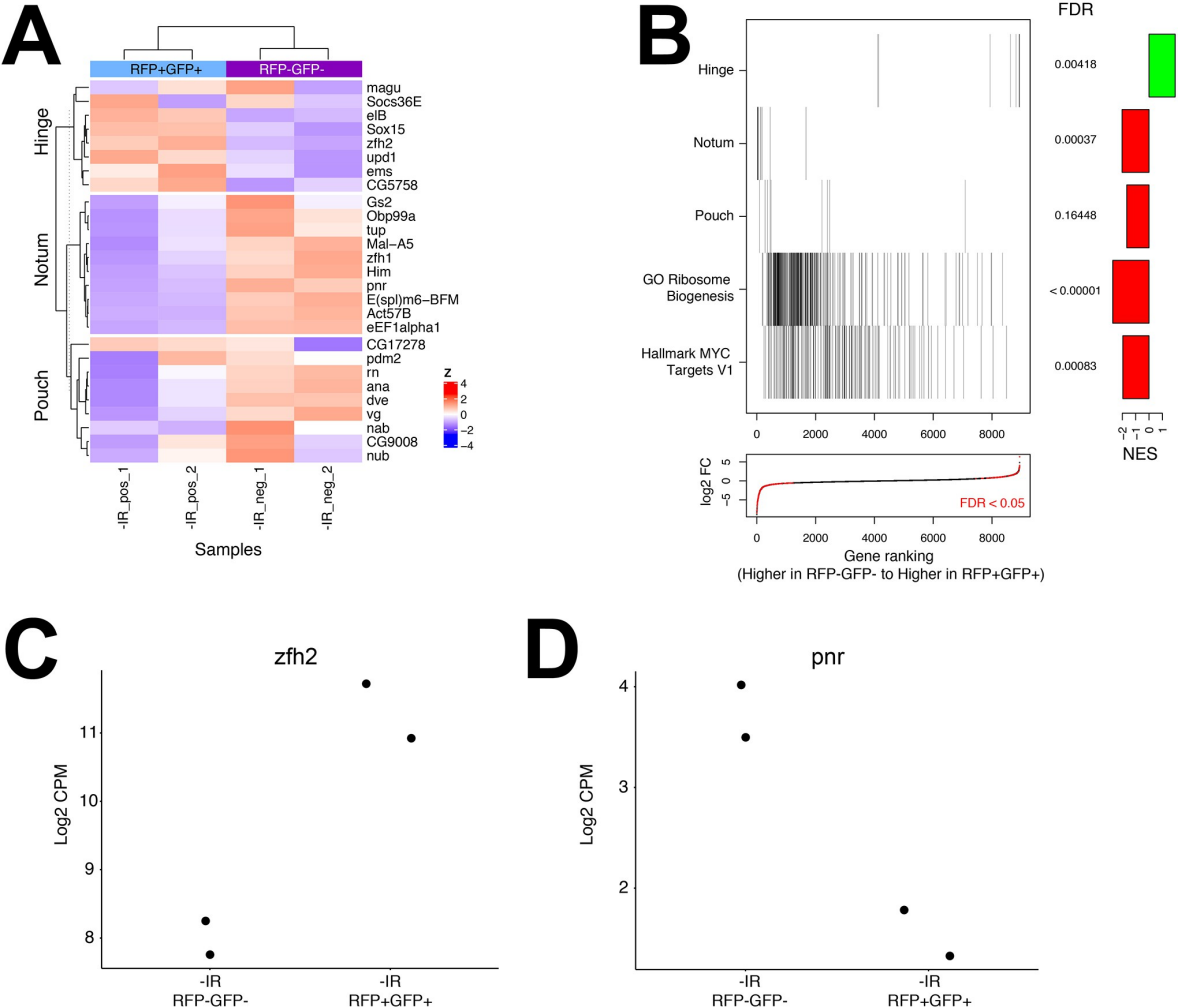
Differential gene expression in POS/pos (RFP^+^GFP^+^) versus NEG/neg (RFP^-^GFP^-^) cells without irradiation. FlyBase nomenclature for gene names is used throughout the manuscript (https://flybase.org). (A) The heatmap shows the expression in POS and NEG cells of genes that are known from the literature to show elevated expression in the hinge, notum, or pouch. (B) Aggregate gene set enrichment scores of the indicated gene sets. Each tick mark represents a gene in the pathway. The barplot shows the normalized enrichment scores (NES, the degree to which a gene set is overrepresented, normalized for gene set size) and p-values adjusted for False Discovery Rate (FDR, estimated probability that a gene set with a given NES represents a false positive finding). See www.gsea-msigdb.org for further definitions. The plot at the bottom shows the fold-changes of genes when comparing POS versus NEG samples in the same order as the pathway data above. Significant (FDR<0.05) genes are highlighted in red. (C-D) The expression in counts per million (CPM) in POS and NEG cells of zfh2 and pnr, representative transcripts with high expression in the hinge and the notum, respectively. Pairs of dots represent data from two biological replicates.

To assess the accuracy of cell sorting procedures, genes known to be differentially expressed in the hinge (8 genes), the pouch (9 genes) or the notum (10 genes) were identified from the literature [20–31], and their basal (-IR) expression was compared between POS and NEG samples (Fig 2A). POS cells showed higher expression of hinge-expressed genes such as *zfh2* (log_2_ = 3.3) [31], *upd1* (log_2_ = 3.6) [26], and *Sox15* (log_2_ = 4.1)[23]. Expression of *ds*, whose sequences drive G-trace, was also ~3 fold higher (log_2_ = 1.8) in the POS cells than in NEG cells. Conversely, NEG cells, which would include both the pouch and the notum, showed higher levels of transcripts known to be primarily or exclusively expressed in the notum such as zfh1 (log_2_ = 4.9), pnr (log_2_ = 2.2), and tup (log_2_ = 1.8) and the pouch such as ana (log_2_ = 2.9), cyp310a1 (log_2_ = 1.6), dve (log_2_ = 1.4) and vg (log_2_ = 1.2) [20,21]. We note that zfh1 is not expressed in the disc proper but in muscle precursors that are associated with the notum [32]. Gene set enrichment analysis [33] showed statistically significant enrichment of hinge gene expression in POS cells and enrichment of notum and pouch gene expression in NEG cells (Fig 2B, top three rows). One representative example each of a gene differentially expressed in POS (*zfh2*) or NEG cells (*pnr*) is shown in Fig 2C and 2D.

As described above, the hinge region of the wing disc differs from the rest of the disc in terms of cellular architecture and regenerative potential [14,15]. To identify additional differences, we asked what functional groups show differential expression between the hinge and the pouch/notum. To our surprise, genes involved in ribosome biogenesis were among the most significant pathways (Fig 2B, 4^th^ row from the top). These are expressed at lower levels in POS cells compared to the NEG cells. A key transcriptional regulator of ribosome biogenesis is the proto-oncogene *myc* [34–36]. Consistent with this, transcriptional targets of Myc are expressed at significantly lower levels in POS cells compared to the NEG cells (Fig 2B, bottom row).

### Gene expression changes following irradiation in POS and NEG cells

**Fig 3:**
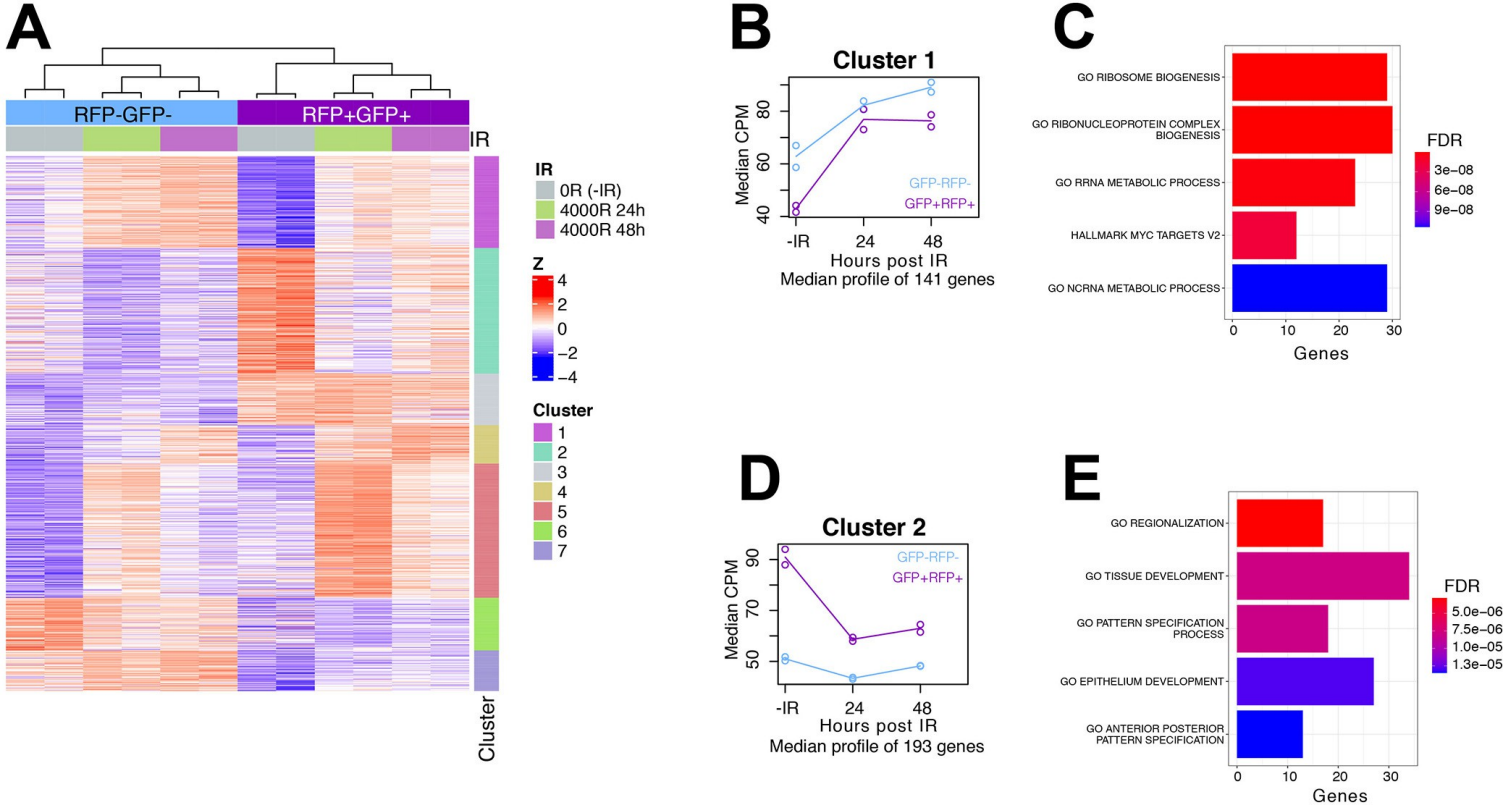
Time series and ORA analysis. (A) Heatmap of 821 significant genes from the time series analysis. Expression data is in S1 Table. Gene names are in S2 Table. (B) Median expression (counts per million) of the genes in Cluster 1 at various treatment and time points. Gene names are in S3 Table. Pairs of circles represent data from two biological replicates. (C) Top five most significant gene sets following ORA for genes in Cluster 1 are shown with FDR-adjusted p-values. Gene names are in S3 Table. (D) Median expression (counts per million) of the genes in Cluster 2 at various treatment and time points. Pairs of circles represent data from two biological replicates. (E) Top five most significant gene sets following ORA for genes in Cluster 2 are shown with FDR-adjusted p-values. Gene names are in S3 Table.

We have shown before that the hinge and the rest of the disc show similar incidence of cells in S and M phases without IR; that is, there are no inherent differences in cell proliferation between these disc regions [14]. Likewise, the hinge and the rest of the disc suffer a similar level of DNA damage after irradiation as detected by γ-H2Av (*Drosophila* γ-H2Ax) staining and show similar kinetics of DNA repair as seen by a time course of disappearance of γ-H2Av [14]. We published IR-induced genome-wide gene expression changes in the whole wing disc and identified changes in genes with roles in DNA damage responses such as DNA repair [37]. We expect these changes to be shared by cells in different parts of the wing disc. The present study is focused not on shared DNA damage responses, which typically occur within 24h after irradiation, but on cell fate changes that occur at later times. Specifically, we are interested in differences in the IR-induced behavior between the hinge and the rest of the disc. To this end, we performed a time course analysis using maSigPro to determine genes whose expression differs between POS and NEG cells following irradiation [38]. This analysis identified 821 genes significantly different across the time course (S1 Table). Gene clustering identified 7 clusters with similar expression patterns (Figs 3A and S2).

### Over-Representation Analysis identified enriched pathways in Clusters 1 and 2

Clustering of the significant genes revealed two clusters of particular interest. Cluster 1 included genes that show lower basal expression in POS cells compared to NEG cells but became more similar after irradiation (Fig 3B). We hypothesize that these genes could encode factors that are upregulated in hinge cells (POS) as they fate-change into pouch cells (NEG), thus making POS cells more like NEG cells. Over Representation Analysis (ORA) of these genes identified pathways involved in ribosome biogenesis and Myc targets (Fig 3C). Cluster 2 included genes that show higher basal expression in POS cells than in NEG cells but became more similar after irradiation (Fig 3D). We hypothesize these genes could encode factors that are downregulated in hinge cells (POS) as they fate-changed into pouch cells (NEG), thus making POS cells more like NEG cells. ORA of these genes identified pathways involved in differentiation-related processes of regionalization, development and specification (Fig 3E). All gene sets with an adjusted p-value <0.05 are shown in S3 Table. Based on these results and other considerations described in S4 Table, 42 genes were tested for functional importance in IR-induced fate change, using overexpression or knock-down approaches. Gene expression changes could drive fate change (drivers) or simply accompany fate change (passengers); therefore, functional tests are needed to tell these apart. Manipulation of six of these genes produced positive data and are described below.

### Functional test of ORA groups: elevation of hinge determinants interfered with fate change

**Fig 4:**
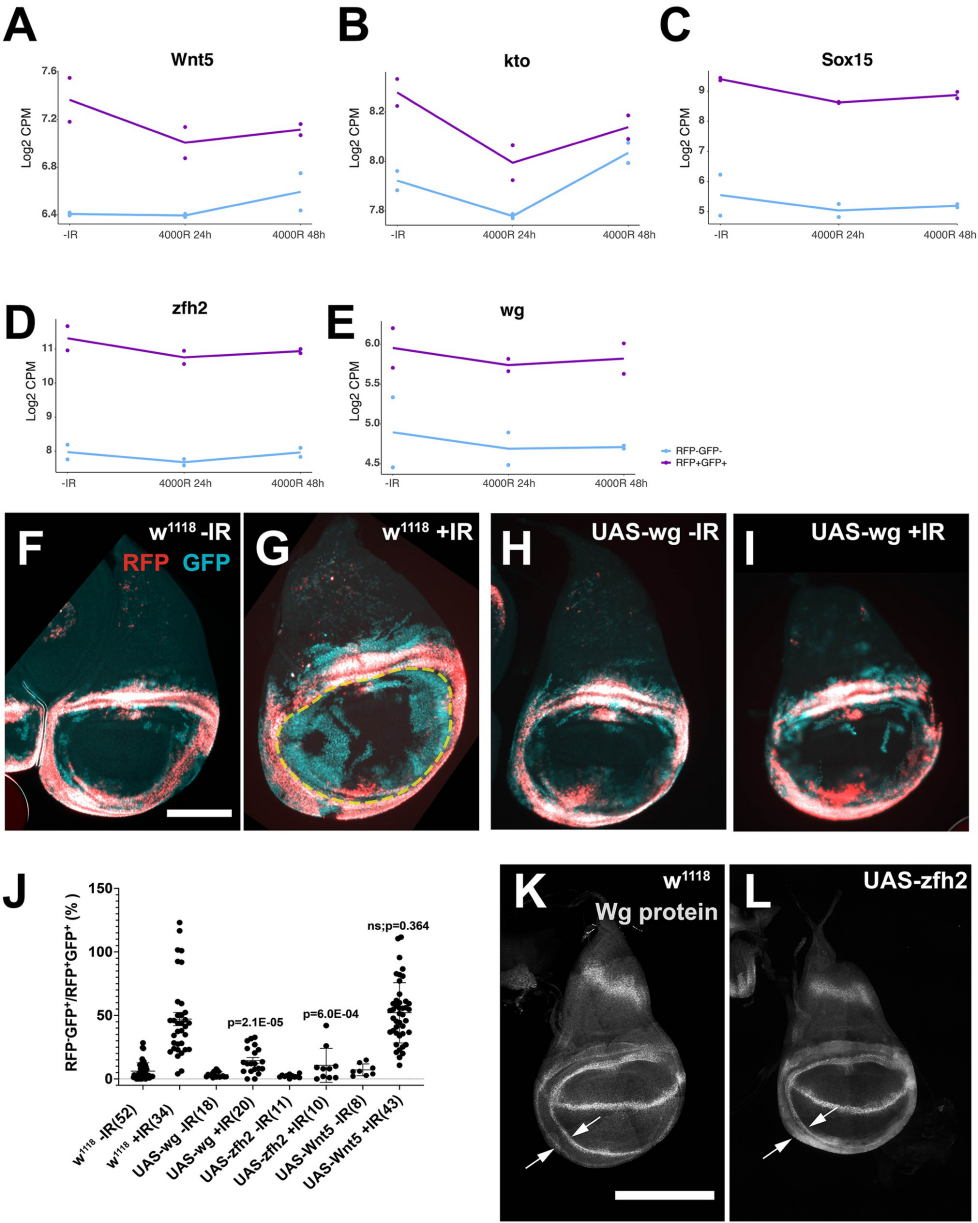
Elevated levels of Wg or Zfh2 inhibit the regenerative behavior of hinge cells. (A-E) Expression trajectories of five genes from the ORA groups on tissue development and specification. Pairs of dots represent data from two biological replicates. Lines connect the averages. (F-I) Wing discs from control and larvae overexpressing UAS-wg treated as shown in Fig 1G. Controls show robust cell fate change and translocation after IR (G) but wg expression in the hinge inhibited IR-induced cell fate plasticity (I). Scale bar = 100 microns. (J) Discs such as those shown in (F-I) were quantified for total RFP^-^GFP^+^ area in the pouch region (within the yellow circle in G) normalized to the RFP^+^GFP^+^ hinge area to account for variations in disc size. Cell autonomous expression of wg and zfh2 but not Wnt5 significantly decreased the normalized RFP^-^GFP^+^ area. p values are for comparison to w^1118^ +IR (2-tailed t-test). The numbers in brackets are the total number of discs examined in two or more biological replicate experiments. (K-L) Antibody staining for Wg protein shows increased signal in the 30A expression domain, for example between two arrows. Scale bar = 100 microns.

Most significant pathways in Cluster 2 included genes with roles in differentiation, suggesting that IR induces a de-differentiated state (Fig 3E and S3 Table). These genes are expressed more highly in the hinge but decreased after IR. Five examples are shown in Fig 4A–4E. The magnitude of the decrease is small in each case, but we note that only a fraction of hinge cells changes fate (Fig 1F’). Zfh2, a transcription factor and an effector in the JAK/STAT pathway, is a key determinant of the hinge fate [31]. kto encodes a homolog of Med12, a subunit of the transcription Mediator complex, and is essential for the expression of downstream targets in the canonical Wnt signaling pathway [39]. Wg and Wnt5 are signaling ligands in the Wnt family and Wg cooperates with Zfh2 in hinge development [31,40,41].

We have reported before that depletion of zfh2 or inhibition of Wg signaling by expressing the inhibitor Axin cell-autonomously in the 30A domain resulted in increased IR-induced apoptosis in the hinge at 4h after irradiation, followed by decreased hinge-to-pouch conversion [12]. The current data suggests that although zfh2 and Wg signaling are required immediately (4h) after irradiation to protect the hinge from IR-induced apoptosis, these factors may need to be downregulated later (24h after IR) for fate change. We tested this hypothesis by conditional overexpression of zfh2, Wg and Wnt5 from UAS-transgenes ([40] and S4 Table).

GAL4 was repressed with GAL80^ts^ and the larvae were allowed to develop to third instar stage at 25°C (Fig 1G). Larvae were shifted to 29°C to inhibit GAL80^ts^ and de-repress GAL4 for 24h before irradiation. Wing discs were analyzed 72h after irradiation and fate change quantified as total area of RFP^-^GFP^+^ cells in the pouch area (within the yellow circle in Fig 4G) normalized to the RFP^+^GFP^+^ hinge to account for variations in disc size, as we have done before [12–14]. The results show that conditional, cell-autonomous overexpression of zfh2 and wg but not Wnt5 reduced fate change and translocation (Fig 4J). These results support the hypothesis that downregulation of zfh2 and Wg signaling is required for cell fate plasticity after irradiation.

Zfh2 functions upstream of Wg expression during normal wing development [31,41]. Therefore, we asked if Zfh2 also functions upstream of Wg during regeneration after irradiation, by staining 30A-GAL4>UAS-zfh2 discs for Wg protein (Fig 4K and 4L). We have shown before that 30A expression domain falls between Wg inner and outer rings (arrows in Fig 4K [14]). In 30A-GAL4>UAS-zfh2 discs, these cells express Wg such that the area between inner and outer rings are ‘filled in’ with Wg antibody stain (between arrows in Fig 4L). Since expression of UAS-wg in the same cells prevented cell fate plasticity, induction of Wg by UAS-zfh2 is one possible way in which UAS-zfh2 prevents cell fate plasticity although each may also be capable of preventing fate change when overexpressed.

### Functional test of ORA groups; inhibition of ribosome biogenesis interfered with fate change

**Fig 5:**
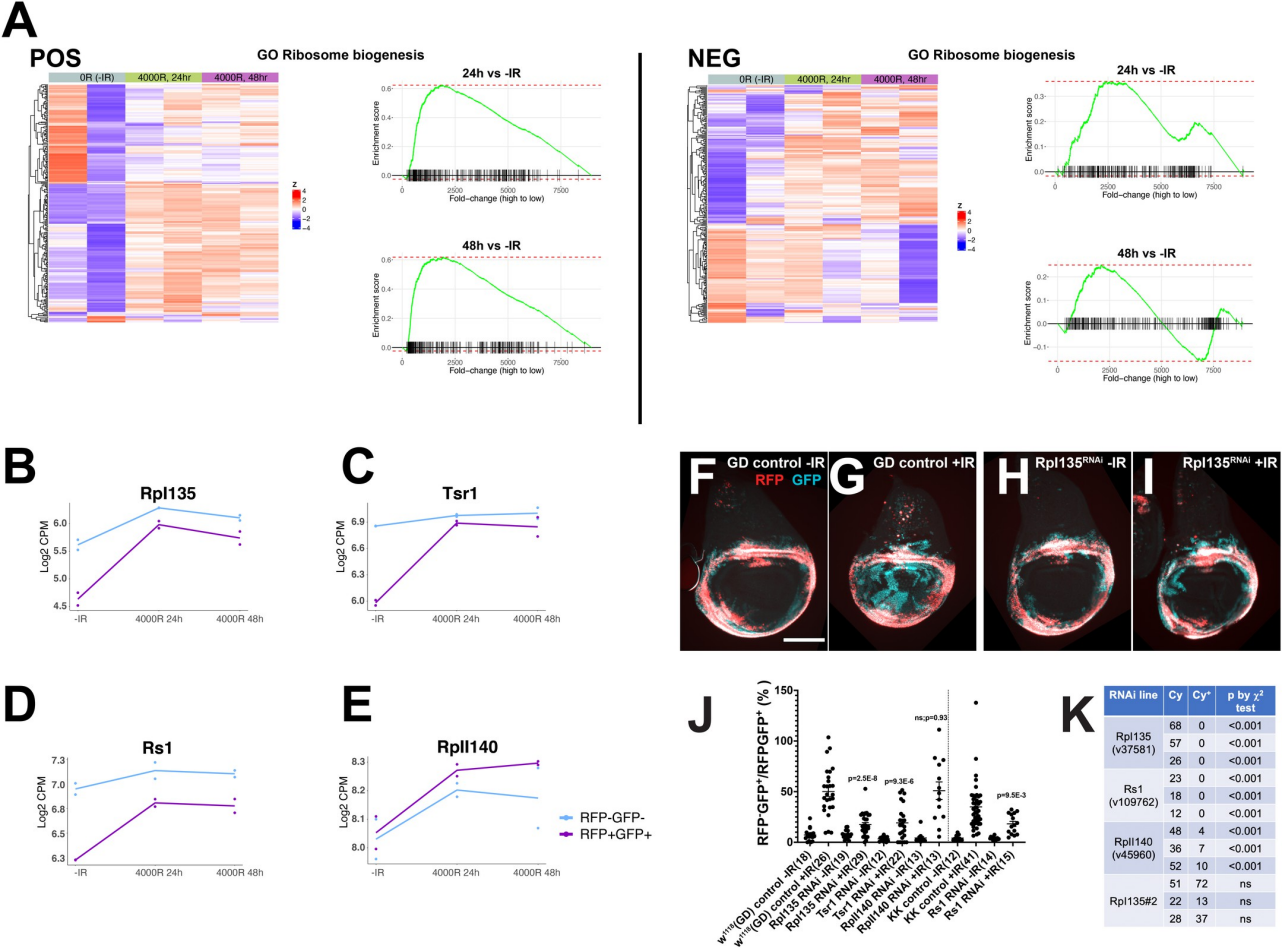
Depletion of ribosome biogenesis factors inhibits the regenerative behavior of hinge cells. (A) Heatmap displaying genes in ribosome biogenesis in POS and NEG cells. Gene names are in S2 Table. Gene set enrichment plots for indicated differential expression comparisons are shown to the side. The fold-change is ranked from high to low, so higher in 24h or 48h compared to -IR. (B-E) Expression trajectory of three ribosome biogenesis genes, RpI135, Rs1 and Tsr1, illustrate how their expression increased to a greater extent in POS cells than in NEG cells after IR. RpII140, encoding a subunit of RNA pol II, is shown here as a counter-example of a gene that does not show this trajectory. Pairs of dots represent data from two biological replicates. Lines connect the averages. (F-I) Wing discs from control larvae with matched background (GD) and larvae expressing RNAi for RpI135 in the hinge, treated as shown in Fig 1G. Controls show robust cell fate change and translocation after IR (G) but RpI135 depletion in the hinge inhibited IR-induced cell fate plasticity (I). Scale bar = 100 microns. (J) Discs such as those shown in (F-I) were quantified for total RFP^-^GFP^+^ area in the pouch area as in Fig 4 and normalized to the RFP^+^GFP^+^ hinge area to account for variations in disc size. Cell autonomous depletion of RpI135, Rs1 and Tsr1 significantly decreased the normalized RFP^-^GFP^+^ area. P values are for comparison to GD+IR (2-tailed t-test). The numbers in brackets are the total number of discs examined in two or more biological replicate experiments. (K) The potency of RNAi constructs was assessed in terms of lethality when constitutively expressed (without GAL80^ts^) from en-GAL4, using the X^2^ test. One parent in each cross was balanced over CyO so that expected ratio if RNAi had no effect was 1 Cy:1Cy^+^. RpI135 (v37581), Rs1 and RpII140 displayed effective RNAi by this measure. The RpI135 RNAi construct #2 did not affect survival and, likewise, did not affect cell fate plasticity (S3 Fig). The data from three independent egg collections for each RNAi line are shown.

**Fig 6:**
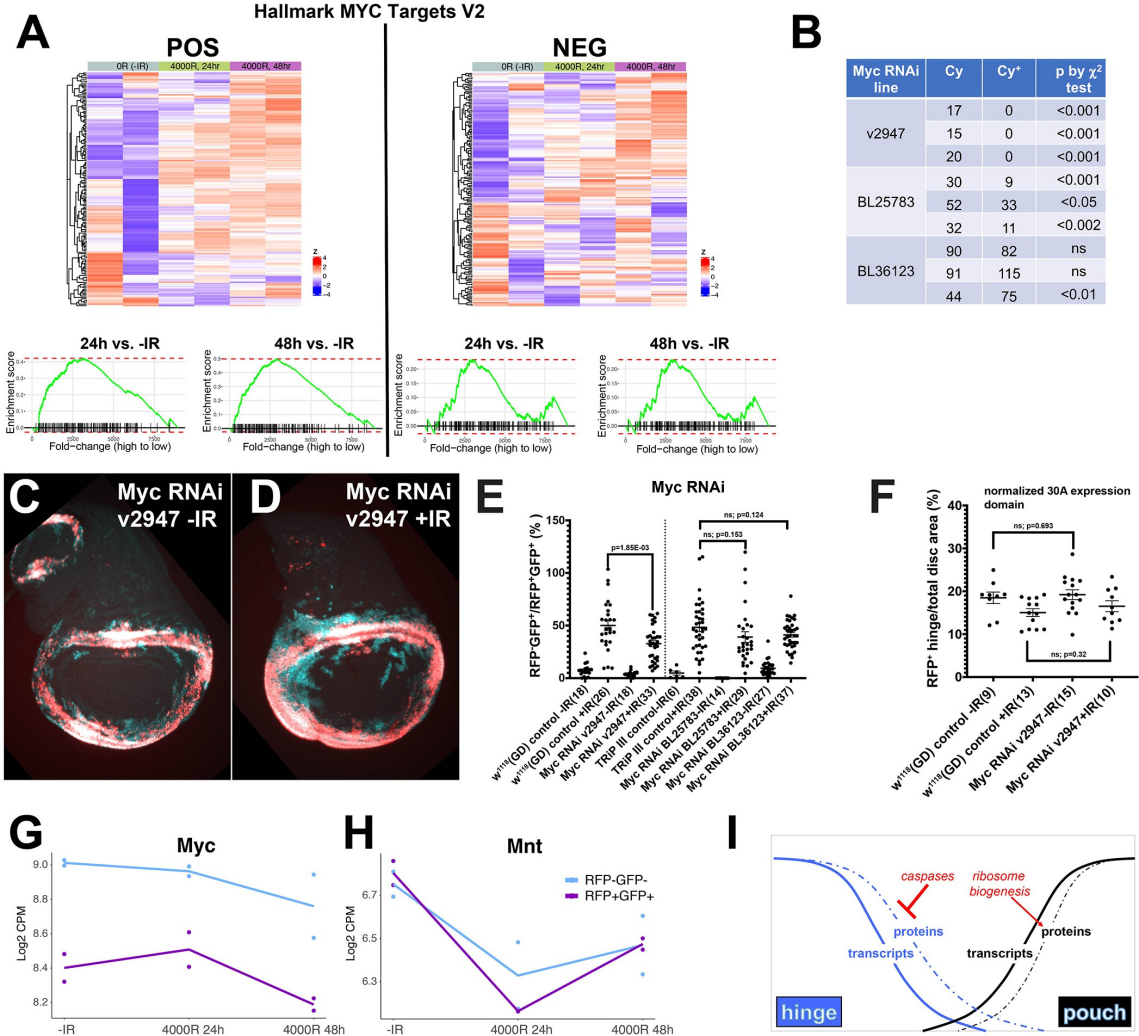
The role of Myc in fate change. (A) Heatmaps displaying Myc targets in POS and NEG cells. Gene names are in S2 Table. Gene set enrichment plots for indicated differential expression comparisons are shown below. The fold-change is ranked from high to low, so higher in 24h or 48h compared to -IR. (B) The potency of RNAi constructs was assessed in terms of lethality when constitutively expressed (without GAL80^ts^) from en-GAL4, using the X^2^ test. One parent in each cross was balanced over CyO so that expected ratio if RNAi had no effect was 1 Cy:1 Cy^+^. v2947 produced complete lethality, BL25783 was partially lethal and BL36123 had no effect. The data from three independent egg collections for each RNAi line are shown. (C-D) Representative wing discs expressing 30A-GAL4>Myc RNAi, with and without IR. (E) Fate change in wing discs from larvae expressing 30A-GAL4>Myc RNAi and treated as in Fig 1G were quantified as in Figs 4 and 5. The results for three RNAi lines were shown next to respective control stocks. Only v2947 reduced fate change significantly. p-values were calculated using a 2-tailed t-test. The numbers in brackets are the total number of discs examined in two or more biological replicate experiments. (F) Under the experimental conditions used to monitor cell fate change, depletion of Myc did not interfere with the formation or growth of the hinge (RFP^+^ domain). p-values were calculated using a 2-tailed t-test. The numbers in brackets are the total number of discs examined in two or more biological replicate experiments. (G-H) Expression trajectories for Myc and its inhibitor Mnt. Pairs of dots represent data from two biological replicates. Lines connect the averages. (I) The model for the role of ribosome biogenesis in fate change.

Among Cluster 1 genes, the most significant ORA group was ribosome biogenesis (Fig 3C and S3 Table). This group of genes shows lower basal expression in the hinge than in the rest of the disc without IR (Fig 2B). After IR, their expression increased in the hinge to a greater degree than in the rest of the disc (Fig 5A). Transcript levels for two such genes, *RpI135* and *Tsr1*, are shown in Fig 5B and 5C. *RpI135* encodes the core subunit of the RNA Polymerase I that transcribes ribosomal RNAs. Tsr1 is the *Drosophila* homolog of a human ribosome assembly factor (FB2021_03 [42]). We identified also Rs1, predicted to encode a nucleolar RNA helicase, as another ribosome assembly function that shows a similar expression profile even though it was placed in Cluster 7 by maSeqPro. We had shown previously that optimal translation is critical for recovery from radiation damage in both *Drosophila* [43] and in mammalian tumor models [44,45]. Specifically, halving the ribosome protein gene dosage or feeding irradiated larvae an inhibitor of translation, bouvardin, reduced survival to adulthood [46]. The current data suggest that translational control may be critical not just for survival but also for IR-induced cell fate changes. To test this hypothesis, we used RNAi to deplete RpI135, Tsr1 and Rs1 (Fig 5F–5I and quantified in 5J). The depletion was conditional, beginning one day before irradiation (Fig 1G), and was cell-autonomous in the 30A domain. By the time of the temperature shift to activate GAL4, the wing discs have developed such that conditional depletion of an essential factor did not affect hinge development (compare Fig 5F and 5H; see also Fig 6F). The results demonstrate that RpI135, Tsr1 and Rs1are needed for IR-induced hinge-to-pouch conversion (Fig 5J).

Comparative analysis of RpII140 helps us rule out the possibility that importance of RpI135, Rs1 and Tsr1 reflects a general requirement for macromolecular synthesis. *RpII140* encodes the large subunit of RNA polymerase II. Unlike RpI135, Rs1 and Tsr1, RpII140 showed neither basal (-IR) differences between POS and NEG samples nor differential expression after IR (Fig 5E). And despite a slight increase in expression after IR in both cell populations (log_2_~0.2), RNAi against RpII140 did not affect IR-induced plasticity (Fig 5J). Although we cannot exclude the possibility of residual protein, this negative result is unlikely to be because of ineffective RNAi; broad expression using an engrailed-GAL4 driver without GAL80 and without irradiation resulted in lethality for RpI135, Rs1 and RpII140 RNAi lines used in Fig 5J (Fig 5K). This is expected as all three genes encode essential functions. In contrast, RpI135 RNAi line#2 provides a counter example of ineffective RNAi; it produced no lethality with en-GAL4 (Fig 5K) and no effect on fate change (S3 Fig). In other words, there is a clear correlation between effective RNAi, as detected independently by organismal lethality without irradiation, and disruption of fate change when conditionally expressed in the hinge. These results suggest collectively that ribosome biogenesis genes are specifically upregulated to a greater extent in the hinge after irradiation and that their function is require for IR-induced hinge-to-pouch conversion.

### Functional test of ORA groups; Myc

An ORA group in Cluster 1 includes Myc targets (Fig 6A, see also Fig 3C and S3 Table). Myc is a key transcriptional regulator of ribosome biogenesis in *Drosophila* and vertebrates [34–36]. RpI135 is a Myc transcriptional target in the *Drosophila* wing disc [47] and is needed for IR-induced fate change (Fig 5I). Therefore, we asked if Myc is also required for IR-induced fate change. We expressed three different Myc RNAi constructs in the hinge conditionally with the 30A-GAL4 driver and quantified the effect on fate change. *myc* is an essential gene; broad and continuous depletion of Myc is expected to cause lethality. Therefore, as for RPI135, we expressed the RNAi constructs broadly and constitutively (with en-GAL4 driver, without GAL80 or IR) and quantified organismal lethality as an independent measure of RNAi efficacy (Fig 6B). Only two RNAi constructs against Myc produced lethality, with one (v2947) showing complete lethality and the other (BL25783) partial lethality. These two are also the constructs that depleted Myc protein (S4 Fig); the third construct, BL36123, had no effect on Myc protein level or lethality (Figs 6B and S4). In fate change assessment, the RNAi construct that produced complete lethality was also the one that inhibited fate change with statistical significance (v2947, Fig 6C–6E). We conclude that effective depletion of Myc inhibited IR-induced fate change. The effect of Myc or ribosome biogenesis on fate change could be because they are needed for fate-changing hinge cells to grow and proliferate as they translocate into the pouch. We do not favor this possibility for the following reason. Although constitutive loss of ribosome function or Myc is expected to inhibit growth and proliferation, conditional depletion of these functions using the protocol in Fig 1G does not appear to affect growth or proliferation; quantification of the RFP^+^ hinge area showed no significant difference between controls and RNAi discs (Fig 6F).

### Additional genes tested

We tested additional genes and pathways based on altered expression in POS cells after IR and because of potential roles in regeneration and cell fate plasticity based on the literature (stock/gene list in S4 Table). None had significant effect on IR-induced fate change (S3 Fig). But we note that we do not know how effective the RNAi/overexpression constructs were under our experimental conditions even though we selected each based on published efficacy (references in S4 Table). For example, a published RNAi construct against TAF1B, another subunit of RNA pol I, was effective in the germline [48] but did not cause lethality when broadly and constitutively expressed using en-GAL4, suggesting it is not effective in somatic cells (S3G Fig); this construct also did not affect IR-induced cell fate change (S3A Fig). Some of the results in S3 Fig may therefore be explained by context-specific ineffectiveness of transgenic constructs.

This concern does not apply to classical loss-of-function mutants. Therefore, it is worth noting that depletion of ribosome function in heterozygotes of *RpL36*^*G0471*^ and *RpS13*^*1*^ did not affect IR-induced fate change (S3B Fig). Heterozygosity in these alleles was shown previously to reduce survival of irradiated larvae into adulthood in our hands, that is, these alleles impair gene function sufficiently to produce a radiation-dependent phenotype [46]. Similarly, systemic reduction of translation with bouvardin, a small molecule inhibitor we have shown previously to reduce the survival of irradiated larvae [46], did not affect IR-induced fate change (S3F Fig) at concentrations that reduces survival. We conclude that while hinge-specific reduction of ribosome biogenesis interfered with IR-induced fate change (Fig 5), systemic reduction of ribosome function did not have the same outcome (S3 Fig). We do not know the reason for this but note that systemic reduction of ribosome function can support life, for example in heterozygotes of ribosomal proteins, but clonal reduction of ribosome function is known to cause death through competition with the neighbors (for a recent review, [49]).

## Discussion

We present here a genome-wide transcriptome analysis of cell fate conversion during regeneration after radiation damage. The ability to trace cells with precise spatial-temporal control, combined with powerful genetic tools to assess gene function in *Drosophila*, allowed us to identify genes needed for IR-induced cell fate plasticity in a cell sub-population of interest. *Drosophila* imaginal discs are composed of a continuous epithelium. An unexpected result is that different regions of this structure express different levels of ribosome biogenesis genes (Fig 2). Irradiation adds another level of change by targeting the same functional group (Fig 5). We are unaware of prior documentation of differential ribosome biogenesis in a continuous epithelium. In fact, ribosomal components are typically used as loading controls to normalize samples across tissues or treatment. Our results question the validity of this practice.

The basal difference in Myc and ribosome biogenesis between the hinge and the rest of the disc is also surprising because such differences, when induced experimentally in neighboring cells, result in cell competition whereby ‘loser’ cells with reduced translation capacity are eliminated via apoptosis (for a recent review, [49]). Despite reduced expression of Myc targets and ribosome biogenesis genes, hinge cells are clearly not ‘losers’ during normal development. But inequalities in translation capacity is just one of many possible triggers for cell competition. The hinge is flanked by two sources of Wg, from Wg Inner and Outer Rings. Elevated Wg signaling is another basis for making the cells ‘winners’ over their neighbors, and this mode of cell competition is independent of Myc [50]. We speculate that reduced ribosome biogenesis may be offset by increased Wg signaling to maintain the hinge during normal development.

Our results suggest that translational capacity increased specifically in the hinge in irradiated discs and that it plays a role in fate change. In human glioblastoma cells, a combination of ribosome profiling and transcriptomics showed that mRNAs that changed ribosome-association were 10-fold greater in number than mRNAs that changed in transcript levels after irradiation [51]. In breast, lung and pancreatic cancer cells, radiation produced profound shifts in ribosome-associated mRNAs [52]. In other words, IR may induce greater changes in translational regulation than transcriptional regulation. Our results advance this knowledge by demonstrating that one outcome of translational changes is IR-induced cell fate plasticity and translocation of cells from the hinge to the pouch. Our results agree with recent findings that increased ribosome biogenesis accompanies Epithelial-Mesenchymal Transition and that pharmacological inhibition of RNA pol I reduced invasiveness and caused de-differentiation (made the tumor more benign) in in vitro and in vivo models of breast cancer [53].

We do not know how irradiation is linked to increased ribosome biogenesis but identify Myc as a potential mediator. In *Drosophila* wing discs that are regenerating after genetic ablation of the pouch, Myc is transcriptionally induced near the wound site [54]. There is surprisingly little in the literature that links Myc and IR, given the central role of Myc in oncology. In our own studies, Myc transcripts show minimal change after IR (Fig 6G and [37]). Interestingly, transcripts for transcription repressor and Myc antagonist Mnt decreased nearly 2-fold after IR in both POS and NEG cells (Fig 6H). Repression of an inhibitor would activate Myc and could link IR with Myc activity.

Elevating the expression of wg or zfh2 interfered with hinge-to-pouch conversion. During wing development, zfh2 is repressed distally along the proximal/distal axis to allow the expression of pouch determinants nab and dve and the formation of the pouch [31,55]. During regeneration, our data suggest that downregulation of zfh2 in the hinge allows hinge-to-pouch transformation, presumably by allowing pouch-specific gene expression as during development. We found also that zfh2 is capable of inducing Wg protein and that elevation of wg on its own prevented fate change. Therefore, induction of Wg provides a second mechanistic explanation for the effect of zfh2 on fate change. The inhibitory role of Wg in fate change in the context of irradiation is seen also in mice where suppression of Wnt signaling prevented ectopic differentiation, maintained the Intestinal Stem Cell pool, and allowed regeneration of the crypt after IR damage [56]. We advance this knowledge with the finding that too much or too little Wg/Zfh2 are equally detrimental. This is because we showed previously that RNAi-mediated depletion of zfh2 or expression of Wg antagonist Axin specifically in the hinge also inhibited hinge-to-pouch conversion after IR [14]. Why might depletion and elevation of wg or zfh2 produce the same outcome? The requirement for wg/zfh2 can be explained by their role in protecting the hinge cells from IR-induced apoptosis, assayed at 4h after irradiation [12,14]. At later times (24-48h after IR), zfh2 and Wg signaling become reduced as cells change fate; elevation of these interfere with fate change (this study), explaining the paradoxical results.

The mechanism by which transcripts of fate determinants are downregulated remains to be investigated. PcG and trxG chromatin complexes are needed for surgically ablated leg discs to change fate or ‘transdetermine’ into wing discs [57,58]. Depletion of Polycomb Repressor Complex 2 members E(z) and Esc using heterozygous mutants, however, produced only negative results on fate change (S3 Fig and S4 Table). Transcription factor tara and a SWI/SNF chromatin-remodeling complex were found to prevent Anterior/Posterior fate switch during regeneration after genetic ablation of the pouch [59,60]. However, *tara*^*1*^ heterozygotes, which produced an A/P switch in the genetic ablation model, did not affect hinge-to-pouch fate change after irradiation (S3B Fig), suggesting different mechanisms for the two fate switch events.

Genome-wide analysis of cell fate conversion after radiation damage reported here add to our published finding that in the wing disc, hinge-to-pouch fate change after irradiation requires apoptotic caspase activity, despite the fact that hinge cells are refractory to IR-induced apoptosis [12]. Collectively, these data support the model that fate change after IR exposure requires not only the induction of new fate determinants but also the removal of old fate determinants (Fig 6I, modified from [61]). This model agrees with the literature that post-transcriptional mechanisms play critical roles in cell fate change (without irradiation) in different systems: miRNAs during *C*. *elegans* development and in mammalian macrophages (to neutralize mRNAs [62–64]) or apoptotic caspases in non-lethal roles during *C*. *elegans* development and in mammalian muscle and embryonic stem cells (to neutralize proteins [65–68]). We add to this knowledge by identifying a new requirement for enhanced translation capacity, presumably to produce proteins that enact the new fate. We plan to focus our future efforts on identifying the mRNA/protein targets of this enhanced translational capacity.

## Materials and methods

### *Drosophila* stocks

Stocks used, along with the sources, genotypes, stock numbers and references are listed in S4 Table. We follow the FlyBase nomenclature for gene names (https://flybase.org). All stocks used are published and/or available from public stock centers. For balanced stocks without a larval marker, the balancers were changed to CyO-GFP or TM6 Tb before use. The absence of the balancer-encoded marker identified the experimental animals. The flies were raised in Nutri-Fly Bloomington formula food (Genesee Scientific) at 25°C unless otherwise noted. Virgins and males were fed Nutri-Fly German formula food (Genesee Scientific) at 25°C for three days after crossing; we found this improves egg production. Embryo collection and subsequent culture were in Nutri-Fly Bloomington formula food. Temperature shift protocols are described in Fig 1. Larval vials were monitored regularly for overcrowding (typically seen as dimples in the food) and split as needed.

### Irradiation and drug treatment

The larvae in food were placed in petri dishes and irradiated in a Faxitron Cabinet X-ray System Model RX-650 (Lincolnshire, IL) at115kv and 5.33rad/second. Bouvardin (NSC259968, NCI DTP) in DMSO was diluted into 5 ml of melted food in vials to achieve the final DMSO concentration of 0.05%. Irradiated larvae were washed clean of food and placed onto the surface of food with bouvardin.

### RNA isolation and processing

We followed a published protocol [69]. Briefly, 30A>G-trace/SM5 virgin females were crossed to w^1118^ males and RFP^+^GFP^+^ larvae were sorted manually. Wing discs were dissected in PBS and dissociated into single cells by limited trypsin digestion as described before RFP^+^GFP^+^ and RFP^-^GFP^-^ cells were collected by Fluorescence-Activated Cell Sorting at the Flow Cytometry Shared Core, University of Colorado at Boulder. Poly-A RNA was isolated using a RNeasy kit (Qiagen) and a KAPA mRNA capture hyper prep kit (Roche) according to manufacturer’s instructions. RNA was sequenced with ERCC spike-ins (ThermoFisher) using a NexSeq 500 sequencer at the BioFrontiers Sequencing Core, University of Colorado at Boulder. To obtain sufficient material for each biological replicate, we pooled discs from multiple independent crosses, dissection and dissociations, and cell sorting runs. Therefore, biological replicates represent independent biological samples, cell lysis, mRNA isolation, and sequence analysis.

### Staining and imaging of wing discs

To collect wing discs, the larvae were dissected in PBS and fixed in 4% paraformaldehyde in PBS for 30 minutes at room temperature, washed with PBS for 10 minutes, followed by a 5 min wash with PBT (0.1% Tween-20). For antibody staining, wing discs were permeabilized in PBT (0.5% Tween-20) for 10’ and rinsed in PBT (0.1% Tween-20). The discs were blocked in 5% Normal Goal Serum in PBT (0.1% Tween-20) for at least 30 min and incubated overnight at 4°C in primary antibody in block (1:100, mouse monoclonal anti-Wg, Developmental Studies Hybridoma Bank Cat# 4D4; undiluted mouse monoclonal anti-Myc, Developmental Studies Hybridoma Bank Cat# P4C4-B10). The discs were rinsed thrice in PBT (0.1% Tween-20) and incubated in secondary antibody at 1:500 dilution in block for 2 h at room temperature. The wing discs were stained with 10μg/mL Hoechst33342 in PBT (0.1% Tween-20) for 2 minutes, washed 3 times in in PBT and mounted on glass slides in Fluromount G (SouthernBiotech). Wing discs were imaged on a Leica DMR compound fluorescence microscope using a Q-Imaging R6 CCD camera and Ocular software. The images were assembled, processed, and quantified using Image J software.

### RNA-sequencing

Illumina adapters and the first 12 base pairs of each read were removed using BBDuk [70] and reads less than 50 base pairs were discarded. STAR (2.6.0a) [71] was used to align and count reads against the Ensembl *Drosophila* melanogaster genome (BDGP6.22, release 97). Differential expression comparing groups was performed using the limma R package and the voom() function [72]. Plots were generated using the ggplot2 R package [73]. The trajectory plots display the log2 counts per million (CPM) for each sample. The line represents the mean of the replicates. Heatmaps of z-score transformed log 2 counts per million were generated using the ComplexHeatmap R package [74]. Samples and genes within each group were hierarchical clustered. Gene set enrichment analysis was performed using the fgsea R package with Hallmark and GO Biological Processes MSigDB gene set collections (V6.2) [75,76]. The pathway enrichment score reflects the magnitude that a pathway is enriched in a given direction in the dataset. The normalized enrichment score (NES) adjusts the enrichment score based on the mean enrichment scores of the dataset. Time course analysis was performed using the maSigPro R package [38]. Genes with a R^2^> = 0.80 and a FDR<0.05 were deemed to be significant. The see.genes() function was used to cluster the genes into 7 clusters. The genes of clusters 1 and 2 were analyzed separately using over-representation analysis (ORA) with the clusterProfiler R package [77] and the gene set collections described above. Raw and processed RNA-seq data are deposited in GEO under accession no. GSE182782.

## Supporting information

S1 Fig A Representative FACS profile of dissociated wing disc cells illustrates the gates used to sort cell populations. FL1 = GFP and FL2 = RFP. R5 represents double positive (RFP^+^GFP^+^) cells and R6 represents double negative (RFP^-^GFP^-^) cells. Gates were set manually using single color (RFP or GFP only) controls and a negative (no fluorescence) control.
(PDF)

S2 Fig Gene clusters identified by maSigPro. 821 genes identified by maSigPro as showing differential expression across the time course were grouped into clusters based on expression patterns. Gene names for each cluster are in S1 Table. Pairs of circles represent data from two biological replicates.
(PDF)

S3 Fig Additional genes tested. (A-F) Experimental conditions and data analysis were as in Fig 4J. Statistical significance was tested against the respective genetic background controls +IR. In (F), the larvae were of the genotype w^1118^/+ or Y; 30A-GAL4>UAS-G-trace/+ produced by a cross between w^1118^ (GD) controls and 30A-GAL4>UAS-G-trace/SM5 and sorted for RFP/GFP. p-values were calculated using a 2-tailed t-test. The data are from two or more biological replicate experiments for each sample. (G) The potency of RNAi constructs was assessed in terms of lethality when constitutively expressed (without GAL80^ts^) from en-GAL4, using the X^2^ test. One parent in each cross was balanced over CyO so that expected ratio if RNAi had no effect was 1 Cy:1 Cy^+^. The data are from three independent egg collections for each RNAi line and suggest that these RNA lines had no effect.
(PDF)

S4 Fig Over-expression and depletion of Myc. Wing discs from 5–6 day old feeding stage larvae were fixed and stained with an antibody against Myc. The discs were imaged and processed identically to allow for comparison of fluorescence signal. (A) A control without primary antibody shows no detectable signal. The outline of the disc from the DNA image is shown. (B) Myc is overexpressed from a UAS transgene in the posterior (P) compartment using the en-GAL4 driver, producing a stronger signal than in the control anterior (A) compartment. The genotype of the larvae was en-GAL4/UAS-Myc. (C-E) Three different RNAi constructs against Myc were expressed in the P compartment and produced different levels of protein depletion. BL36123 produced no discernable difference between A and P compartments. v2947 and BL25783 reduced the Myc signal in the posterior (P, arrows) compared to the anterior. The genotype of the larvae was en-GAL4/UAS-RNAi or en-GAL4/+; UAS-RNAi/+. See S4 Table for more information on transgenic stocks.
(PDF)

S1 Table 821 significant genes from time series analysis using maSeqPro. Clusters, p-values, and counts per million (CPM) are provided.
(TXT)

S2 Table Genes shown in heatmaps in Figs 3, 5 and 6 are listed in the order in which they appear from the top of the heatmap. (TXT)

S3 Table Over-Representation Analysis (ORA) of the genes in Clusters 1 and 2. Only gene sets with an adjusted p<0.05 are shown. Cluster 1 is called ‘neg_high.up’ because genes in this cluster show higher basal expression in NEG cells over POS cells but increased in expression in the latter after irradiation. Cluster 2 is called ‘pos_high.down’ because genes in this cluster show higher basal expression in POS cells than in NEG cells but decreased in expression in the former after irradiation.
(TXT)

S4 Table Genes tested for their role in IR-induced hinge-to-pouch conversion, along with the stock information and additional citations. (XLSX)

S1 Data Raw data for Figs 4–6 are provided as a Prism file. (PZFX)

S2 Data Raw data for S3 Fig are provided as a Prism file. (PZFX)

## References

[1] Marjanovic ND, Weinberg RA, Chaffer CL. Cell plasticity and heterogeneity in cancer. Clin Chem. 2013;59(1):168–79. doi: 10.1373/clinchem.2012.184655 . 23220226 PMC6220421

[2] Ye X, Weinberg RA. Epithelial-Mesenchymal Plasticity: A Central Regulator of Cancer Progression. Trends Cell Biol. 2015;25(11):675–86. doi: 10.1016/j.tcb.2015.07.012 ; PubMed Central PMCID: PMC4628843. 26437589 PMC4628843

[3] Gomez KE, Wu F, Keysar SB, Morton JJ, Miller B, Chimed TS, et al. Cancer Cell CD44 Mediates Macrophage/Monocyte-Driven Regulation of Head and Neck Cancer Stem Cells. Cancer Res. 2020;80(19):4185–98. Epub 2020/08/21. doi: 10.1158/0008-5472.CAN-20-1079 ; PubMed Central PMCID: PMC8146866. 32816856 PMC8146866

[4] Keysar SB, Le PN, Miller B, Jackson BC, Eagles JR, Nieto C, et al. Regulation of Head and Neck Squamous Cancer Stem Cells by PI3K and SOX2. J Natl Cancer Inst. 2017;109(1). Epub 2016/09/17. doi: 10.1093/jnci/djw189 ; PubMed Central PMCID: PMC5025278. 27634934 PMC5025278

[5] Keysar SB, Jimeno A. More than markers: biological significance of cancer stem cell-defining molecules. Mol Cancer Ther. 2010;9(9):2450–7. Epub 2010/08/19. doi: 10.1158/1535-7163.MCT-10-0530 ; PubMed Central PMCID: PMC3618879. 20716638 PMC3618879

[6] Lagadec C, Vlashi E, Della Donna L, Dekmezian C, Pajonk F. Radiation-induced reprogramming of breast cancer cells. Stem Cells. 2012;30(5):833–44. doi: 10.1002/stem.1058 ; PubMed Central PMCID: PMC3413333. 22489015 PMC3413333

[7] Lee SY, Jeong EK, Ju MK, Jeon HM, Kim MY, Kim CH, et al. Induction of metastasis, cancer stem cell phenotype, and oncogenic metabolism in cancer cells by ionizing radiation. Mol Cancer. 2017;16(1):10. doi: 10.1186/s12943-016-0577-4 ; PubMed Central PMCID: PMC5282724. 28137309 PMC5282724

[8] Pisco AO, Huang S. Non-genetic cancer cell plasticity and therapy-induced stemness in tumour relapse: ’What does not kill me strengthens me’. Br J Cancer. 2015;112(11):1725–32. doi: 10.1038/bjc.2015.146 ; PubMed Central PMCID: PMC4647245. 25965164 PMC4647245

[9] Vlashi E, Chen AM, Boyrie S, Yu G, Nguyen A, Brower PA, et al. Radiation-Induced Dedifferentiation of Head and Neck Cancer Cells Into Cancer Stem Cells Depends on Human Papillomavirus Status. Int J Radiat Oncol Biol Phys. 2016;94(5):1198–206. doi: 10.1016/j.ijrobp.2016.01.005 ; PubMed Central PMCID: PMC4817367. 27026319 PMC4817367

[10] Yu S, Tong K, Zhao Y, Balasubramanian I, Yap GS, Ferraris RP, et al. Paneth Cell Multipotency Induced by Notch Activation following Injury. Cell Stem Cell. 2018;23(1):46–59 e5. Epub 2018/06/12. doi: 10.1016/j.stem.2018.05.002 ; PubMed Central PMCID: PMC6035085. 29887318 PMC6035085

[11] Weng PL, Aure MH, Maruyama T, Ovitt CE. Limited Regeneration of Adult Salivary Glands after Severe Injury Involves Cellular Plasticity. Cell Rep. 2018;24(6):1464–70 e3. Epub 2018/08/09. doi: 10.1016/j.celrep.2018.07.016 ; PubMed Central PMCID: PMC6350767. 30089258 PMC6350767

[12] Verghese S, Su TT. Ionizing radiation induces stem cell-like properties in a caspase-dependent manner in Drosophila. PLoS Genet. 2018;14(11):e1007659. Epub 2018/11/22. doi: 10.1371/journal.pgen.1007659 ; PubMed Central PMCID: PMC6248896. 30462636 PMC6248896

[13] Verghese S, Su TT. STAT, Wingless, and Nurf-38 determine the accuracy of regeneration after radiation damage in Drosophila. PLoS Genet. 2017;13(10):e1007055. Epub 2017/10/14. doi: 10.1371/journal.pgen.1007055 ; PubMed Central PMCID: PMC5656321. 29028797 PMC5656321

[14] Verghese S, Su TT. Drosophila Wnt and STAT Define Apoptosis-Resistant Epithelial Cells for Tissue Regeneration after Irradiation. PLoS Biol. 2016;14(9):e1002536. Epub 2016/09/02. doi: 10.1371/journal.pbio.1002536 ; PubMed Central PMCID: PMC5008734. 27584613 PMC5008734

[15] Tamori Y, Suzuki E, Deng WM. Epithelial Tumors Originate in Tumor Hotspots, a Tissue-Intrinsic Microenvironment. PLoS Biol. 2016;14(9):e1002537. Epub 2016/09/02. doi: 10.1371/journal.pbio.1002537 ; PubMed Central PMCID: PMC5008749. 27584724 PMC5008749

[16] Herrera SC, Martin R, Morata G. Tissue homeostasis in the wing disc of Drosophila melanogaster: immediate response to massive damage during development. PLoS Genet. 2013;9(4):e1003446. Epub 2013/05/02. doi: 10.1371/journal.pgen.1003446 ; PubMed Central PMCID: PMC3636033. 23633961 PMC3636033

[17] Myat MM, Louis D, Mavrommatis A, Collins L, Mattis J, Ledru M, et al. Regulators of cell movement during development and regeneration in Drosophila. Open Biol. 2019;9(5):180245. Epub 2019/05/02. doi: 10.1098/rsob.180245 ; PubMed Central PMCID: PMC6544984. 31039676 PMC6544984

[18] Clark HF, Brentrup D, Schneitz K, Bieber A, Goodman C, Noll M. Dachsous encodes a member of the cadherin superfamily that controls imaginal disc morphogenesis in Drosophila. Genes Dev. 1995;9(12):1530–42. Epub 1995/06/15. doi: 10.1101/gad.9.12.1530 7601355

[19] Evans CJ, Olson JM, Ngo KT, Kim E, Lee NE, Kuoy E, et al. G-TRACE: rapid Gal4-based cell lineage analysis in Drosophila. Nat Methods. 2009;6(8):603–5. Epub 2009/07/28. doi: 10.1038/nmeth.1356 ; PubMed Central PMCID: PMC2754220. 19633663 PMC2754220

[20] Butler MJ, Jacobsen TL, Cain DM, Jarman MG, Hubank M, Whittle JR, et al. Discovery of genes with highly restricted expression patterns in the Drosophila wing disc using DNA oligonucleotide microarrays. Development. 2003;130(4):659–70. Epub 2002/12/31. doi: 10.1242/dev.00293 . 12505997

[21] Calleja M, Herranz H, Estella C, Casal J, Lawrence P, Simpson P, et al. Generation of medial and lateral dorsal body domains by the pannier gene of Drosophila. Development. 2000;127(18):3971–80. Epub 2000/08/23. . 10952895 10.1242/dev.127.18.3971

[22] Cifuentes FJ, Garcia-Bellido A. Proximo-distal specification in the wing disc of Drosophila by the nubbin gene. Proc Natl Acad Sci U S A. 1997;94(21):11405–10. Epub 1997/10/23. doi: 10.1073/pnas.94.21.11405 PubMed Central PMCID: PMC23481. 9326622 PMC23481

[23] Cremazy F, Berta P, Girard F. Genome-wide analysis of Sox genes in Drosophila melanogaster. Mech Dev. 2001;109(2):371–5. Epub 2001/12/04. doi: 10.1016/s0925-4773(01)00529-9 . 11731252

[24] Everetts NJ, Worley MI, Yasutomi R, Yosef N, Hariharan IK. Single-cell transcriptomics of the Drosophila wing disc reveals instructive epithelium-to-myoblast interactions. Elife. 2021;10. Epub 2021/03/23. doi: 10.7554/eLife.61276 ; PubMed Central PMCID: PMC8021398. 33749594 PMC8021398

[25] Jacobsen TL, Cain D, Paul L, Justiniano S, Alli A, Mullins JS, et al. Functional analysis of genes differentially expressed in the Drosophila wing disc: role of transcripts enriched in the wing region. Genetics. 2006;174(4):1973–82. Epub 2006/10/10. doi: 10.1534/genetics.106.056788 ; PubMed Central PMCID: PMC1698657. 17028348 PMC1698657

[26] Johnstone K, Wells RE, Strutt D, Zeidler MP. Localised JAK/STAT pathway activation is required for Drosophila wing hinge development. PLoS One. 2013;8(5):e65076. Epub 2013/06/07. doi: 10.1371/journal.pone.0065076 ; PubMed Central PMCID: PMC3669132. 23741461 PMC3669132

[27] Karsten P, Hader S, Zeidler MP. Cloning and expression of Drosophila SOCS36E and its potential regulation by the JAK/STAT pathway. Mech Dev. 2002;117(1–2):343–6. Epub 2002/09/03. doi: 10.1016/s0925-4773(02)00216-2 . 12204282

[28] Lai EC, Bodner R, Posakony JW. The enhancer of split complex of Drosophila includes four Notch-regulated members of the bearded gene family. Development. 2000;127(16):3441–55. Epub 2000/07/21. . 10903170 10.1242/dev.127.16.3441

[29] St Pierre SE, Galindo MI, Couso JP, Thor S. Control of Drosophila imaginal disc development by rotund and roughened eye: differentially expressed transcripts of the same gene encoding functionally distinct zinc finger proteins. Development. 2002;129(5):1273–81. Epub 2002/03/05. . 11874922 10.1242/dev.129.5.1273

[30] Terriente Felix J, Magarinos M, Diaz-Benjumea FJ. Nab controls the activity of the zinc-finger transcription factors Squeeze and Rotund in Drosophila development. Development. 2007;134(10):1845–52. Epub 2007/04/13. doi: 10.1242/dev.003830 . 17428824

[31] Terriente J, Perea D, Suzanne M, Diaz-Benjumea FJ. The Drosophila gene zfh2 is required to establish proximal-distal domains in the wing disc. Dev Biol. 2008;320(1):102–12. Epub 2008/06/24. doi: 10.1016/j.ydbio.2008.04.028 . 18571155

[32] Boukhatmi H, Bray S. A population of adult satellite-like cells in Drosophila is maintained through a switch in RNA-isoforms. Elife. 2018;7. Epub 2018/04/10. doi: 10.7554/eLife.35954 ; PubMed Central PMCID: PMC5919756. 29629869 PMC5919756

[33] Sergushichev AA. An algorithm for fast preranked gene set enrichment analysis using cumulative statistic calculation. 2016.

[34] Destefanis F, Manara V, Bellosta P. Myc as a Regulator of Ribosome Biogenesis and Cell Competition: A Link to Cancer. Int J Mol Sci. 2020;21(11). Epub 2020/06/11. doi: 10.3390/ijms21114037 ; PubMed Central PMCID: PMC7312820. 32516899 PMC7312820

[35] van Riggelen J, Yetil A, Felsher DW. MYC as a regulator of ribosome biogenesis and protein synthesis. Nat Rev Cancer. 2010;10(4):301–9. Epub 2010/03/25. doi: 10.1038/nrc2819 . 20332779

[36] Dai MS, Lu H. Crosstalk between c-Myc and ribosome in ribosomal biogenesis and cancer. J Cell Biochem. 2008;105(3):670–7. Epub 2008/09/06. doi: 10.1002/jcb.21895 ; PubMed Central PMCID: PMC2569974. 18773413 PMC2569974

[37] van Bergeijk P, Heimiller J, Uyetake L, Su TT. Genome-wide expression analysis identifies a modulator of ionizing radiation-induced p53-independent apoptosis in Drosophila melanogaster. PLoS One. 2012;7(5):e36539. Epub 2012/06/06. doi: 10.1371/journal.pone.0036539 ; PubMed Central PMCID: PMC3362589. 22666323 PMC3362589

[38] Nueda MJ, Tarazona S, Conesa A. Next maSigPro: updating maSigPro bioconductor package for RNA-seq time series. Bioinformatics. 2014;30(18):2598–602. Epub 2014/06/05. doi: 10.1093/bioinformatics/btu333 ; PubMed Central PMCID: PMC4155246. 24894503 PMC4155246

[39] Carrera I, Janody F, Leeds N, Duveau F, Treisman JE. Pygopus activates Wingless target gene transcription through the mediator complex subunits Med12 and Med13. Proc Natl Acad Sci U S A. 2008;105(18):6644–9. Epub 2008/05/03. doi: 10.1073/pnas.0709749105 ; PubMed Central PMCID: PMC2373359. 18451032 PMC2373359

[40] Lawrence PA, Sanson B, Vincent JP. Compartments, wingless and engrailed: patterning the ventral epidermis of Drosophila embryos. Development. 1996;122(12):4095–103. Epub 1996/12/01. 9012529 10.1242/dev.122.12.4095

[41] Perea D, Terriente J, Diaz-Benjumea FJ. Temporal and spatial windows delimit activation of the outer ring of wingless in the Drosophila wing. Dev Biol. 2009;328(2):445–55. Epub 2009/02/17. doi: 10.1016/j.ydbio.2009.02.002 . 19217893

[42] Larkin A, Marygold SJ, Antonazzo G, Attrill H, Dos Santos G, Garapati PV, et al. FlyBase: updates to the Drosophila melanogaster knowledge base. Nucleic Acids Res. 2021;49(D1):D899–D907. Epub 2020/11/22. doi: 10.1093/nar/gkaa1026 ; PubMed Central PMCID: PMC7779046. 33219682 PMC7779046

[43] Jaklevic B, Uyetake L, Lemstra W, Chang J, Leary W, Edwards A, et al. Contribution of growth and cell cycle checkpoints to radiation survival in Drosophila. Genetics. 2006;174(4):1963–72. Epub 2006/10/10. doi: 10.1534/genetics.106.064477 ; PubMed Central PMCID: PMC1698627. 17028317 PMC1698627

[44] Stickel SA, Gomes NP, Frederick B, Raben D, Su TT. Bouvardin is a Radiation Modulator with a Novel Mechanism of Action. Radiat Res. 2015;184(4):392–403. Epub 2015/09/29. doi: 10.1667/RR14068.1 ; PubMed Central PMCID: PMC4643058. 26414509 PMC4643058

[45] Keysar SB, Gomes N, Miller B, Jackson BC, Le PN, Morton JJ, et al. Inhibiting Translation Elongation with SVC112 Suppresses Cancer Stem Cells and Inhibits Growth in Head and Neck Squamous Carcinoma. Cancer Res. 2020;80(5):1183–98. Epub 2020/01/09. doi: 10.1158/0008-5472.CAN-19-3232 ; PubMed Central PMCID: PMC7056512. 31911553 PMC7056512

[46] Gladstone M, Frederick B, Zheng D, Edwards A, Yoon P, Stickel S, et al. A translation inhibitor identified in a Drosophila screen enhances the effect of ionizing radiation and taxol in mammalian models of cancer. Dis Model Mech. 2012;5(3):342–50. Epub 2012/02/22. doi: 10.1242/dmm.008722 ; PubMed Central PMCID: PMC3339828. 22344740 PMC3339828

[47] Grewal SS, Li L, Orian A, Eisenman RN, Edgar BA. Myc-dependent regulation of ribosomal RNA synthesis during Drosophila development. Nat Cell Biol. 2005;7(3):295–302. Epub 2005/02/22. doi: 10.1038/ncb1223 . 15723055

[48] Zhang Q, Shalaby NA, Buszczak M. Changes in rRNA transcription influence proliferation and cell fate within a stem cell lineage. Science. 2014;343(6168):298–301. Epub 2014/01/18. doi: 10.1126/science.1246384 ; PubMed Central PMCID: PMC4084784. 24436420 PMC4084784

[49] Baker NE. Emerging mechanisms of cell competition. Nat Rev Genet. 2020;21(11):683–97. Epub 2020/08/12. doi: 10.1038/s41576-020-0262-8 ; PubMed Central PMCID: PMC8205513. 32778819 PMC8205513

[50] Vincent JP, Kolahgar G, Gagliardi M, Piddini E. Steep differences in wingless signaling trigger Myc-independent competitive cell interactions. Dev Cell. 2011;21(2):366–74. Epub 2011/08/16. doi: 10.1016/j.devcel.2011.06.021 ; PubMed Central PMCID: PMC3209557. 21839923 PMC3209557

[51] Lu X, de la Pena L, Barker C, Camphausen K, Tofilon PJ. Radiation-induced changes in gene expression involve recruitment of existing messenger RNAs to and away from polysomes. Cancer Res. 2006;66(2):1052–61. Epub 2006/01/21. doi: 10.1158/0008-5472.CAN-05-3459 . 16424041

[52] Kumaraswamy S, Chinnaiyan P, Shankavaram UT, Lu X, Camphausen K, Tofilon PJ. Radiation-induced gene translation profiles reveal tumor type and cancer-specific components. Cancer Res. 2008;68(10):3819–26. Epub 2008/05/17. doi: 10.1158/0008-5472.CAN-08-0016 ; PubMed Central PMCID: PMC2553206. 18483266 PMC2553206

[53] Prakash V, Carson BB, Feenstra JM, Dass RA, Sekyrova P, Hoshino A, et al. Ribosome biogenesis during cell cycle arrest fuels EMT in development and disease. Nat Commun. 2019;10(1):2110. Epub 2019/05/10. doi: 10.1038/s41467-019-10100-8 ; PubMed Central PMCID: PMC6506521. 31068593 PMC6506521

[54] Smith-Bolton RK, Worley MI, Kanda H, Hariharan IK. Regenerative growth in Drosophila imaginal discs is regulated by Wingless and Myc. Dev Cell. 2009;16(6):797–809. Epub 2009/06/18. doi: 10.1016/j.devcel.2009.04.015 ; PubMed Central PMCID: PMC2705171. 19531351 PMC2705171

[55] Ayala-Camargo A, Anderson AM, Amoyel M, Rodrigues AB, Flaherty MS, Bach EA. JAK/STAT signaling is required for hinge growth and patterning in the Drosophila wing disc. Dev Biol. 2013;382(2):413–26. Epub 2013/08/28. doi: 10.1016/j.ydbio.2013.08.016 ; PubMed Central PMCID: PMC3795806. 23978534 PMC3795806

[56] Gregorieff A, Liu Y, Inanlou MR, Khomchuk Y, Wrana JL. Yap-dependent reprogramming of Lgr5(+) stem cells drives intestinal regeneration and cancer. Nature. 2015;526(7575):715–8. Epub 2015/10/28. doi: 10.1038/nature15382 . 26503053

[57] Worley MI, Setiawan L, Hariharan IK. Regeneration and transdetermination in Drosophila imaginal discs. Annu Rev Genet. 2012;46:289–310. Epub 2012/09/01. doi: 10.1146/annurev-genet-110711-155637 . 22934642

[58] Klebes A, Sustar A, Kechris K, Li H, Schubiger G, Kornberg TB. Regulation of cellular plasticity in Drosophila imaginal disc cells by the Polycomb group, trithorax group and lama genes. Development. 2005;132(16):3753–65. Epub 2005/08/04. doi: 10.1242/dev.01927 . 16077094

[59] Schuster KJ, Smith-Bolton RK. Taranis Protects Regenerating Tissue from Fate Changes Induced by the Wound Response in Drosophila. Dev Cell. 2015;34(1):119–28. Epub 2015/06/23. doi: 10.1016/j.devcel.2015.04.017 . 26096735

[60] Tian Y, Smith-Bolton RK. Regulation of growth and cell fate during tissue regeneration by the two SWI/SNF chromatin-remodeling complexes of Drosophila. Genetics. 2021;217(1):1–16. Epub 2021/03/09. doi: 10.1093/genetics/iyaa028 ; PubMed Central PMCID: PMC8045688. 33683366 PMC8045688

[61] Su TT. Cellular plasticity, caspases and autophagy; that which does not kill us, well, makes us different. Open Biol. 2018;8(11). Epub 2018/11/30. doi: 10.1098/rsob.180157 ; PubMed Central PMCID: PMC6282069. 30487302 PMC6282069

[62] Lee RC, Feinbaum RL, Ambros V. The C. elegans heterochronic gene lin-4 encodes small RNAs with antisense complementarity to lin-14. Cell. 1993;75(5):843–54. Epub 1993/12/03. doi: 10.1016/0092-8674(93)90529-y 8252621

[63] Reinhart BJ, Slack FJ, Basson M, Pasquinelli AE, Bettinger JC, Rougvie AE, et al. The 21-nucleotide let-7 RNA regulates developmental timing in Caenorhabditis elegans. Nature. 2000;403(6772):901–6. Epub 2000/03/08. doi: 10.1038/35002607 . 10706289

[64] Rodriguez-Ubreva J, Ciudad L, van Oevelen C, Parra M, Graf T, Ballestar E. C/EBPa-mediated activation of microRNAs 34a and 223 inhibits Lef1 expression to achieve efficient reprogramming into macrophages. Mol Cell Biol. 2014;34(6):1145–57. Epub 2014/01/15. doi: 10.1128/MCB.01487-13 ; PubMed Central PMCID: PMC3958044. 24421386 PMC3958044

[65] Dick SA, Chang NC, Dumont NA, Bell RA, Putinski C, Kawabe Y, et al. Caspase 3 cleavage of Pax7 inhibits self-renewal of satellite cells. Proc Natl Acad Sci U S A. 2015;112(38):E5246–52. Epub 2015/09/16. doi: 10.1073/pnas.1512869112 ; PubMed Central PMCID: PMC4586827. 26372956 PMC4586827

[66] Wang H, Loof S, Borg P, Nader GA, Blau HM, Simon A. Turning terminally differentiated skeletal muscle cells into regenerative progenitors. Nat Commun. 2015;6:7916. Epub 2015/08/06. doi: 10.1038/ncomms8916 ; PubMed Central PMCID: PMC4765497. 26243583 PMC4765497

[67] Fujita J, Crane AM, Souza MK, Dejosez M, Kyba M, Flavell RA, et al. Caspase activity mediates the differentiation of embryonic stem cells. Cell Stem Cell. 2008;2(6):595–601. Epub 2008/06/05. doi: 10.1016/j.stem.2008.04.001 ; PubMed Central PMCID: PMC2494585. 18522852 PMC2494585

[68] Weaver BP, Zabinsky R, Weaver YM, Lee ES, Xue D, Han M. CED-3 caspase acts with miRNAs to regulate non-apoptotic gene expression dynamics for robust development in C. elegans. Elife. 2014;3:e04265. Epub 2014/11/29. doi: 10.7554/eLife.04265 ; PubMed Central PMCID: PMC4279084. 25432023 PMC4279084

[69] Khan SJ, Abidi SN, Tian Y, Skinner A, Smith-Bolton RK. A rapid, gentle and scalable method for dissociation and fluorescent sorting of imaginal disc cells for mRNA sequencing. Fly (Austin). 2016;10(2):73–80. Epub 2016/04/09. doi: 10.1080/19336934.2016.1173296 ; PubMed Central PMCID: PMC4934706. 27057746 PMC4934706

[70] Bushnell B. BB Tools: BBMap 2021. Available from: https://sourceforge.net/projects/bbmap/.

[71] Dobin A, Davis CA, Schlesinger F, Drenkow J, Zaleski C, Jha S, et al. STAR: ultrafast universal RNA-seq aligner. Bioinformatics. 2013;29(1):15–21. Epub 2012/10/30. doi: 10.1093/bioinformatics/bts635 ; PubMed Central PMCID: PMC3530905. 23104886 PMC3530905

[72] Ritchie ME, Phipson B, Wu D, Hu Y, Law CW, Shi W, et al. limma powers differential expression analyses for RNA-sequencing and microarray studies. Nucleic Acids Res. 2015;43(7):e47. Epub 2015/01/22. doi: 10.1093/nar/gkv007 ; PubMed Central PMCID: PMC4402510. 25605792 PMC4402510

[73] Wickham H. ggplot2: Elegant Graphics for Data Analysis. New York: Springer-Verlag 2016.

[74] Gu Z, Eils R, Schlesner M. Complex heatmaps reveal patterns and correlations in multidimensional genomic data. Bioinformatics. 2016;32(18):2847–9. Epub 2016/05/22. doi: 10.1093/bioinformatics/btw313 . 27207943

[75] Dolgalev I. msigdbr: MSigDB Gene Sets for Multiple Organisms in a Tidy Data Format. 2020. R package version 7.2.1.: [Available from: https://CRAN.R-project.org/package=msigdbr.

[76] Subramanian A, Tamayo P, Mootha VK, Mukherjee S, Ebert BL, Gillette MA, et al. Gene set enrichment analysis: a knowledge-based approach for interpreting genome-wide expression profiles. Proc Natl Acad Sci U S A. 2005;102(43):15545–50. Epub 2005/10/04. doi: 10.1073/pnas.0506580102 ; PubMed Central PMCID: PMC1239896. 16199517 PMC1239896

[77] Yu G, Wang LG, Han Y, He QY. clusterProfiler: an R package for comparing biological themes among gene clusters. OMICS. 2012;16(5):284–7. Epub 2012/03/30. doi: 10.1089/omi.2011.0118 ; PubMed Central PMCID: PMC3339379. 22455463 PMC3339379

[78] Dietzl G, Chen D, Schnorrer F, Su KC, Barinova Y, Fellner M, et al. A genome-wide transgenic RNAi library for conditional gene inactivation in Drosophila. Nature. 2007;448(7150):151–6. Epub 2007/07/13. doi: 10.1038/nature05954 . 17625558

