# Supplementary figures and images for: Differential gene expression analysis identified determinants of cell fate plasticity during radiation-induced regeneration in Drosophila
Source: PLoS Genet. 2022 Jan 6;18(1):e1009989. doi: 10.1371/journal.pgen.1009989 (PMC8769364; doi:10.1371/journal.pgen.1009989)

Supplemental Figure 1

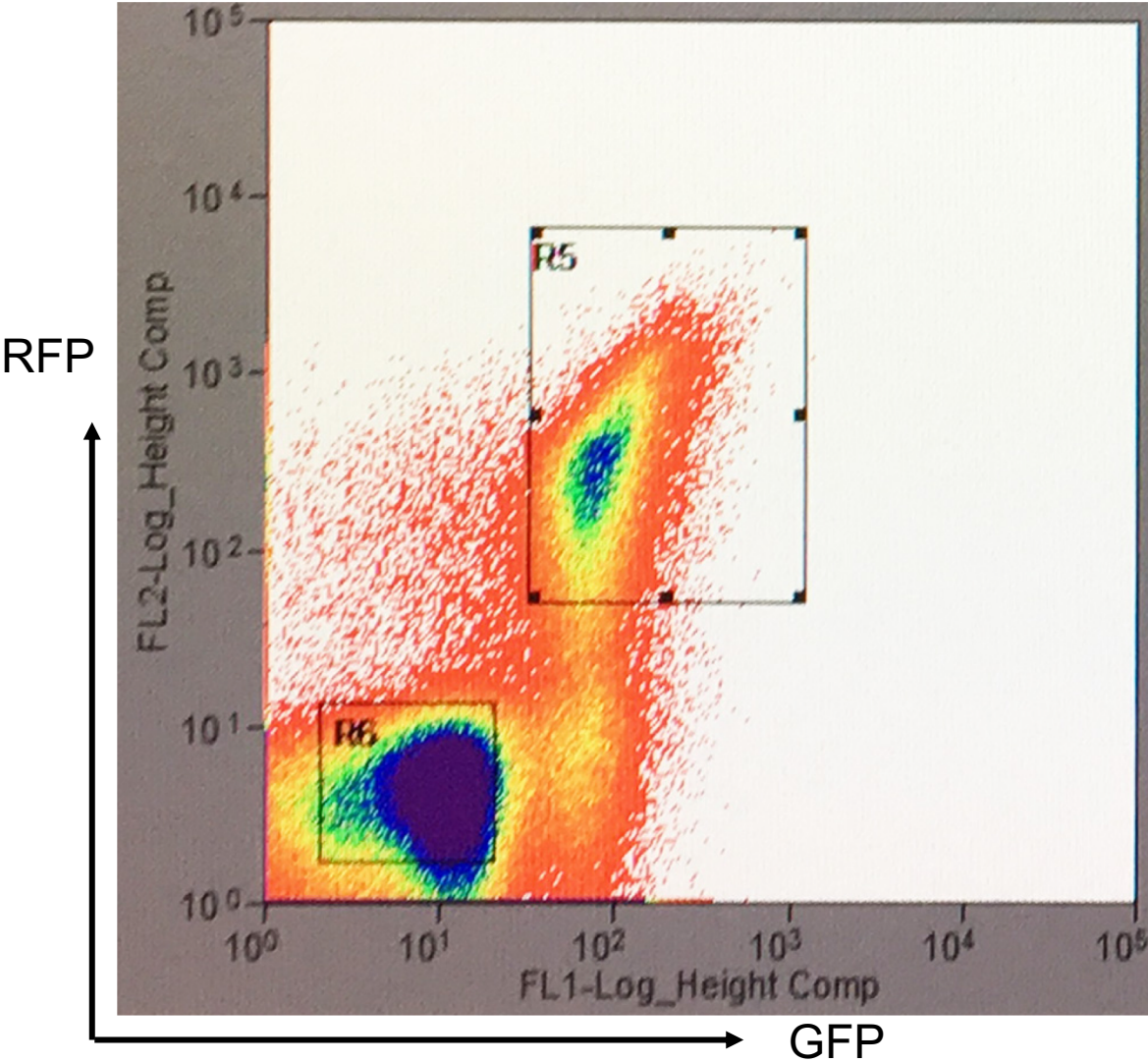

Supplement: S1 Fig — FL1 = GFP and FL2 = RFP. R5 represents double positive (RFP+GFP+) cells and R6 represents double negative (RFP-GFP-) cells. Gates were set manually using single color (RFP or GFP only) controls and a negative (no fluorescence) control. (PDF) [file pgen.1009989.s001.pdf]

Supplemental Figure 2

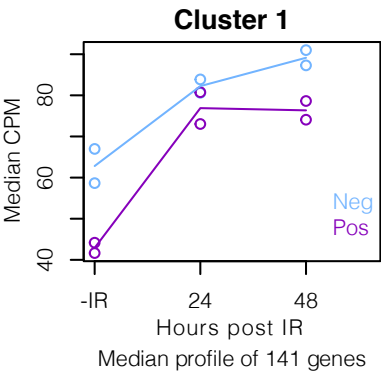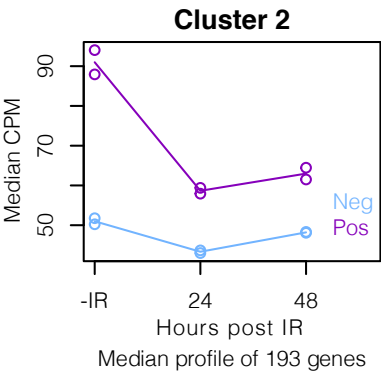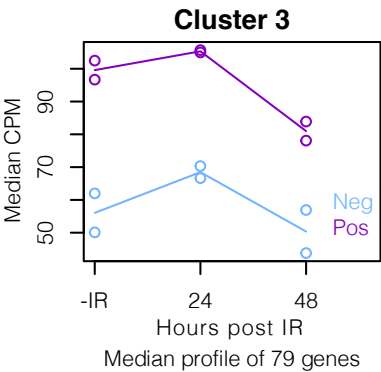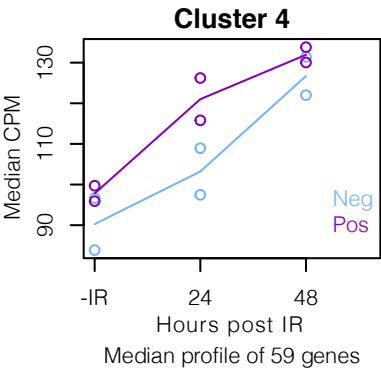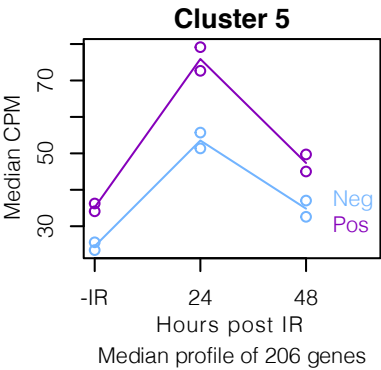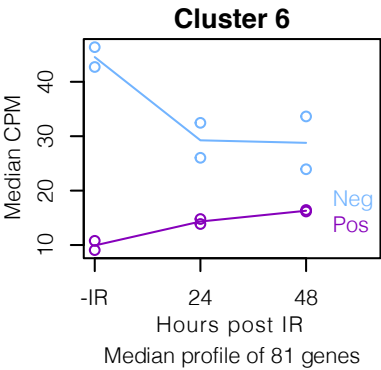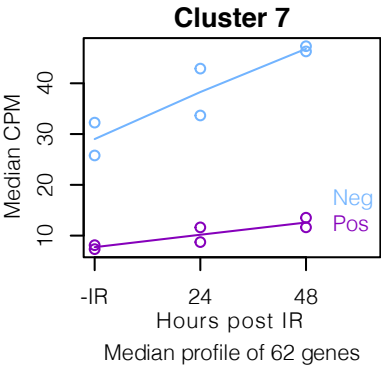

Supplement: S2 Fig — 821 genes identified by maSigPro as showing differential expression across the time course were grouped into clusters based on expression patterns. Gene names for each cluster are in S1 Table. Pairs of circles represent data from two biological replicates. (PDF) [file pgen.1009989.s002.pdf]

Supplemental Figure 3

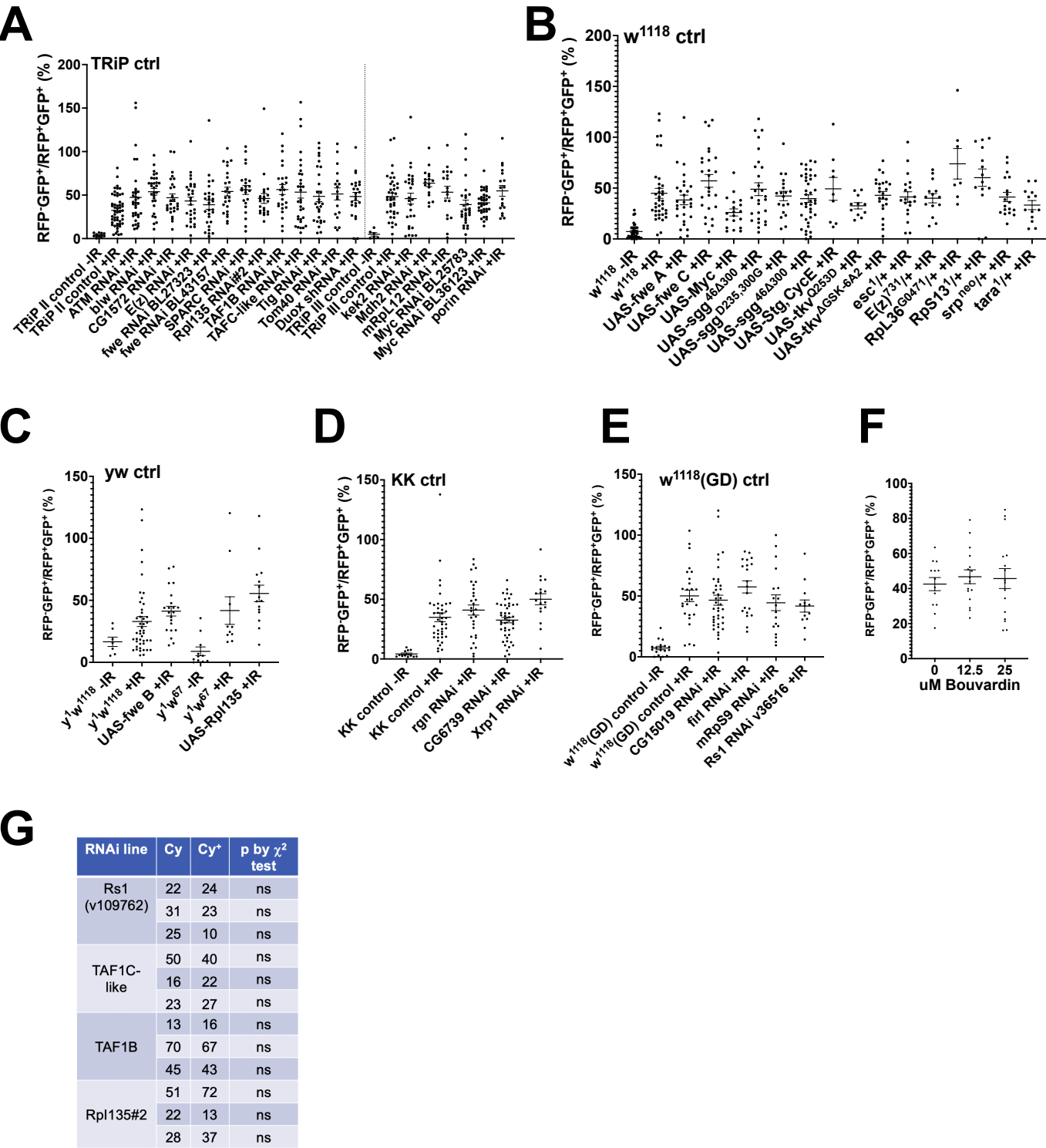

Supplement: S3 Fig — (A-F) Experimental conditions and data analysis were as in Fig 4J. Statistical significance was tested against the respective genetic background controls +IR. In (F), the larvae were of the genotype w1118/+ or Y; 30A-GAL4>UAS-G-trace/+ produced by a cross between w1118 (GD) controls and 30A-GAL4>UAS-G-trace/SM5 and sorted for RFP/GFP. p-values were calculated using a 2-tailed t-test. The data are from two or more biological replicate experiments for each sample. (G) The potency of RNAi constructs was assessed in terms of lethality when constitutively expressed (without GAL80ts) from en-GAL4, using the X2 test. One parent in each cross was balanced over CyO so that expected ratio if RNAi had no effect was 1 Cy:1 Cy+. The data are from three independent egg collections for each RNAi line and suggest that these RNA lines had no effect. (PDF) [file pgen.1009989.s003.pdf]

Supplemental Figure 4

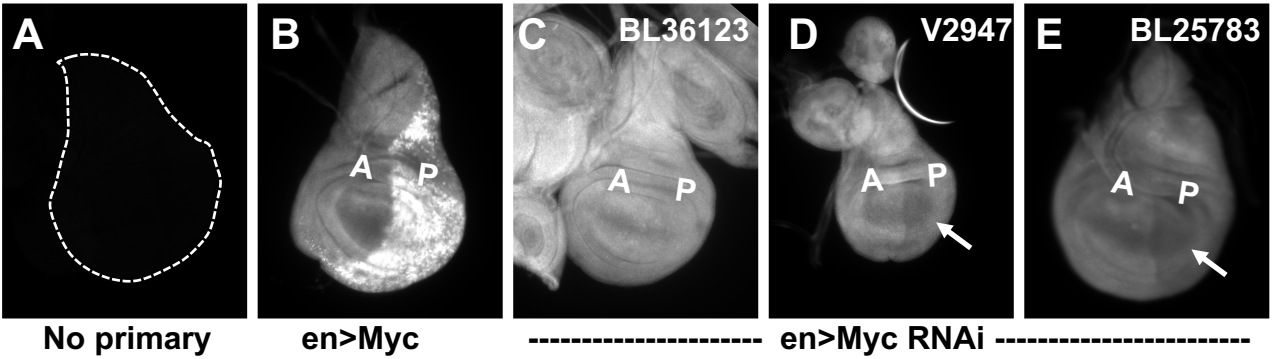

Supplement: S4 Fig — Wing discs from 5–6 day old feeding stage larvae were fixed and stained with an antibody against Myc. The discs were imaged and processed identically to allow for comparison of fluorescence signal. (A) A control without primary antibody shows no detectable signal. The outline of the disc from the DNA image is shown. (B) Myc is overexpressed from a UAS transgene in the posterior (P) compartment using the en-GAL4 driver, producing a stronger signal than in the control anterior (A) compartment. The genotype of the larvae was en-GAL4/UAS-Myc. (C-E) Three different RNAi constructs against Myc were expressed in the P compartment and produced different levels of protein depletion. BL36123 produced no discernable difference between A and P compartments. v2947 and BL25783 reduced the Myc signal in the posterior (P, arrows) compared to the anterior. The genotype of the larvae was en-GAL4/UAS-RNAi or en-GAL4/+; UAS-RNAi/+. See S4 Table for more information on transgenic stocks. (PDF) [file pgen.1009989.s004.pdf]
